# Crystal structure of the lipopolysaccharide outer core galactosyltransferase WaaB involved in pathogenic bacterial invasion of host cells

**DOI:** 10.3389/fmicb.2023.1239537

**Published:** 2023-09-22

**Authors:** Yatian Chen, Jiayue Gu, Gareth Ashworth, Zhongshan Wang, Zhengyu Zhang, Changjiang Dong

**Affiliations:** ^1^School of Pharmaceutical Sciences, Wuhan University, Wuhan, China; ^2^Key Laboratory of Combinatorial Biosynthesis and Drug Discovery, Ministry of Education, School of Pharmaceutical Sciences, Wuhan University, Wuhan, China; ^3^Biomedical Research Centre, Norwich Medical School, University of East Anglia, Norwich Research Park, Norwich, United Kingdom; ^4^Jiangsu Province Key Laboratory of Anesthesiology, Xuzhou Medical University, Xuzhou, China

**Keywords:** lipopolysaccharide synthesis, WaaB, X-ray crystallography, galactosyltransferase, bacterial invasion, structural microbiology

## Abstract

Lipopolysaccharide (LPS) is essential for most gram-negative bacteria and plays an important role in serum resistance, pathogenesis, drug resistance, and protection from harsh environments. The outer core oligosaccharide of LPS is involved in bacterial recognition and invasion of host cells. The D-galactosyltransferase WaaB is responsible for the addition of D-galactose to the outer core oligosaccharide of LPS, which is essential for *Salmonella typhimurium* invasion. Here we report the first crystal structures of WaaB and WaaB in complex with UDP to resolutions of 1.8 and 1.9 Å, respectively. Mutagenesis and enzyme activity assays confirmed that residues V186, K195, I216, W243, E276, and E269 of WaaB are essential for the binding and hydrolysis of UDP-galactose. The elucidation of the catalytic mechanism of WaaB is of great importance and could potentially be used for the design of novel therapeutic reagents.

## Introduction

**Figure 1:**
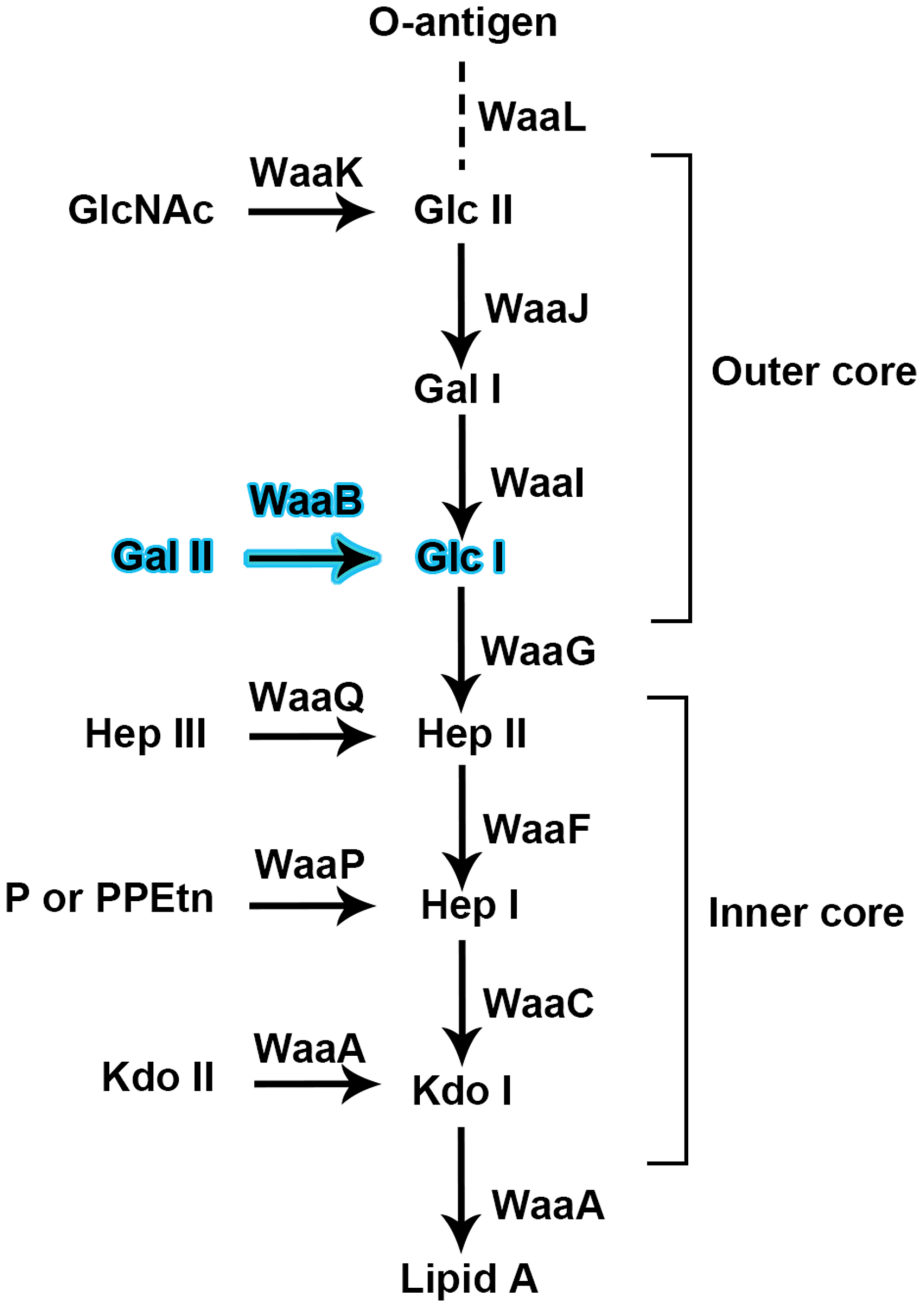
Glycosyltransferases are proposed for the biosynthesis of the inner core and outer core of the lipopolysaccharide of *Salmonella typhimurium* strain LT2. WaaB is responsible for transfer D-galactose from the donor substrate UDP-galactose to the acceptor substrate Glc-Hep_2_-1-dephospho-Kdo_2_-lipid A.

Lipopolysaccharide (LPS) is an essential component of the outer leaflet of the outer membrane (OM) in most Gram-negative bacteria, helping to protect the bacteria from hostile environments and antibiotics (Ashraf et al., 2022). LPS contains three moieties: lipid A (the hydrophobic membrane anchor), core oligosaccharide (a nonrepeating oligosaccharide), and O-antigen (a polymer of a repeating oligosaccharide unit). The core oligosaccharide can be further divided into two structurally distinct regions: the inner core and the outer core (Figure 1). Both core regions play important roles in the survival and virulence of gram-negative bacteria.

The gram-negative bacterium *S. typhimurium* causes serious diseases in humans and a wide variety of farm animals (Control and Prevention, 2003; Galan, 2021), resulting in more than 1 billion infections worldwide each year (Hoare et al., 2006; Crump et al., 2015). The impermeable outer membrane of *S. typhimurium* provides resistance against a large number of noxious agents, contributing to *S. typhimurium* being such a destructive pathogen. The O-antigen portion of LPS has been recognized as an important virulence determinant in the pathogenesis of systemic salmonellosis (Okuda et al., 2016; Simpson and Trent, 2019). Recently increasing amounts of research suggest that the core oligosaccharide of LPS also contribute to bacterial virulence (Hoare et al., 2006; Clifton et al., 2016). In particular, the core oligosaccharide of LPS have been reported to be involved in host cell recognition and invasion (Hoare et al., 2006; Wang et al., 2021).

The biosynthesis of the outer core region of the *S. typhimurium* LPS requires the sequential addition of sugars to a growing polysaccharide chain (Figure 1); the glycosyltransferases (GTs) responsible for this process are located in the *rfa*GB1J gene cluster (Wang and Quinn, 2010). In *S. typhimurium*, the gene product of *waaB*, also named *rfaB*, is an α1, 6-linked D-galactosyltransferase which transfers the D-galactose residue from UDP-galactose to the polysaccharide core (Glc-Hep_2_-1-dephospho-Kdo_2_-lipid A). The *waaB* deletion mutant lacks the α1, 6-linked D-galactose residue of the core LPS. It leads to the attenuation of virulence in *S. typhimurium* (Morgan et al., 2004; Hoare et al., 2006) and significantly reduces bacterial interaction with, and invasion of epithelial cells. In addition, the *waaB* deletion strain was observed to be less tolerate to bile salts, suggesting that the residue added by *waaB* to the core of LPS contributes to the detergent resistance of *S. typhimurium* (Su et al., 2009; Ruan et al., 2012; Qian et al., 2014).

WaaB belongs to the glycosyltransferase family 4 (GT4 family), which is the largest family of glycosyltransferases and also contains several enzymes of therapeutic interest. This diverse family contains examples of enzymes that not only utilize the nucleotide-sugar donors, but also simple phospho-sugars and lipid-phospho-sugar donors, hinting at the ancient origins of this family (Schmid et al., 2016; Zhang et al., 2020). Sharing functional similarity with other LPS biosynthesis-related GTs, WaaB should possess a nucleotide-sugar (UDP-galactose) binding site (donor-substrate nucleotide-sugar binding site), a hydrolytically active site and a potential acceptor substrate binding site. This GT family includes many GTs involved in LPS oligosaccharide biosynthesis (Martinez-Fleites et al., 2006; Williams et al., 2017). Interestingly, despite the folding and functional similarity of related LPS glycosyltransferases, WaaB shares less than 17% sequence identity with these enzymes, suggesting that WaaB holds relatively unique features. There are around 13 Waa proteins involved in the biosynthesis of the core oligosaccharide of LPS, but only three crystal structures of WaaA, WaaC, and WaaG have been reported (Schmidt et al., 2012; Pagnout et al., 2019). WaaA, WaaC, and WaaG are responsible for the addition of 3-deoxy-D-manno-oct-ulosonic acid (Kdo), L-glycero-D-manno-heptose (heptose), and glucose from the CMP-Kdo, ADP-L-glycero-β-D-mano-heptose and UDP-glucose to the LPS core oligosaccharide, respectively.

Here, we present the first X-ray crystal structure of WaaB, providing insight into the addition of galactose to the outer core of LPS oligosaccharide, which shows the structural specificity of donor substrate binding and acceptor substrate binding. Our glycosyltransferase activity assay identifies the catalytic residues involved in UDP-galactose hydrolysis and illustrates the importance of a hydrophobic pocket for UDP-galactose binding.

## Materials and methods

### Cloning, expression, and purification of *Salmonella typhimurium* WaaB

Gene (*rfaB*) fragment encoding WaaB from *S. typhimurium* strain LT2 was amplified by PCR and ligated into pLOU3 (Amp^R^) which expresses the target protein as a fusion protein that contains at the N terminus: hexa-histidine tag-Maltose binding protein (MBP)-tobacco etch virus (TEV) protease cleavage site-*S. typhimurium* WaaB. The recombinant plasmid was transformed into BL21 (DE3) strain (Novagen) for protein expression. The expression cells were cultured in Luria Bertani (LB) medium supplemented with antibiotic (ampicillin 100 μg/mL) at 37°C for 1.5 to 2 h until the optical density of the culture measured at a wavelength of 600 nm reached 0.6–0.8. Isopropyl β-D-Thiogalactoside (IPTG) was added to the medium at a final concentration of 0.1 mM to induce the fusion protein. After expressing the recombinant protein for 4 h at 37°C, cells were harvested at 6,000 × g for 15 min. The cell pellet was re-suspended in buffer (20 mM Tris-Cl, pH 8.0, 300 mM NaCl, 10 mM imidazole, and 10% glycerol), supplemented with EDTA-free protease inhibitor tablet (Roche) and 50 μg/mL DNase I (Sigma-Aldrich). The cells were lysed by passing through a cell disruptor at 30,000 psi (Constant Systems Ltd). Cell debris was removed by centrifugation at 18,000 × g for 30 min at 4°C. The supernatant was loaded into 5 mL HisTrap HP (Ge Healthcare) and washed with 100 mL wash buffer (20 mM Tris-Cl, pH 8.0, 300 mM NaCl, 30 mM imidazole, and 10% glycerol). The fusion protein was eluted with 20 mM Tris-Cl, pH 8.0, 300 mM NaCl, 300 mM imidazole, and 10% glycerol. The fusion protein was then desalted with 20 mM Tris-Cl, pH 8.0, 300 mM NaCl, 10% glycerol, and 10 mM imidazole using a desalting column (Hi-PrepTM 26/10, GE Healthcare) to remove the high concentration of imidazole for next step of TEV protease treatment. After overnight treatment with TEV protease at room temperature, MBP together with hexa-histidine tag was removed, and the samples were then passed through a 5 mL HisTrap HP to isolate WaaB from the mixture and collect the flow-through samples containing WaaB. The protein was further purified by size exclusion chromatography using a HiLoad 16/60 Superdex 200 prep grade column (GE Healthcare) in buffer (20 mM Tris-Cl, pH 8.0 and 150 mM NaCl). The highest purity protein fraction was collected and concentrated to 10 mg/mL for crystallization experiments.

### Crystallization and data collection

WaaB crystallization was carried out by mixing 0.8 μL of protein with 0.8 μL of reservoir solution by the sitting-drop vapor diffusion technique at room temperature. The best crystals of *S. typhimurium* WaaB were obtained in 1% trypton, 1 mM sodium azide, 50 mM HEPES, pH 7.0, and 20% w/v PEG 3350. The crystals appeared within 14 days and grew to full size in 28 days. The soaking of sodium iodide (NaI) into crystals was carried out by soaking the crystals into a buffer containing 1 M NaI, 50 mM HEPES, pH 7.0, 20% w/v PEG 3350, and 20% glycerol. The crystals were also soaked with UDP-galactose in the cryoprotectant buffer containing 100 mM UDP-galactose. Apo crystals and crystals in complex with UDP-galactose were flash frozen in liquid nitrogen after being cryoprotected by the cryoprotectant buffer or soaking buffer. The diffraction data for crystals of WaaB apo and WaaB in complex with UDP-galactose were collected at Diamond Light Source beamline I02 (United Kingdom) and Shanghai Synchrotron Radiation Facility beamline BL19U1 (China) at a wavelength of 0.97949 Å. The Single-wavelength anomalous dispersion data (SAD) for NaI was collected at a wavelength of 1.82330 Å. The *S. typhimurium* WaaB data was processed to 1.9 Å using XDS (Kabsch, 2010). The crystals belonged to space group *P*4_1_2_1_2 with unit-cell dimensions: *a* = 104.09 Å, *b* = 104.09 Å, *c* = 89 Å, and *α* = *β* = *γ* = 90°. There was one molecule per asymmetric unit (Table 1).

**Table 1:** Statistics of data collection and refinement of *S. typhimurium* WaaB.

Data collection	*S. typhimurium* native WaaB	*S. typhimurium* UDP-WaaB
Resolution (Å)	67.64–1.81 (1.85–1.81)	73.85–1.92 (1.97–1.92)
Wavelength (Å)	1.823	0.979
Space Group	P4_3_2_1_2	P4_3_2_1_2
Completeness (%)	98.5 (86.2)	99.9 (99.5)
I/sigma	29.0 (3.1)	16.1 (1.9)
Multiplicity	60.1 (18.2)	23.4 (11.8)
CC1/2 (%)	96 (62)	98 (54)
Unique reflections	44,340 (2800)	38,460 (2767)
R_merge_ (%)	5.3 (12.5)	7.6 (16)
Unite Cell (Å)	*a* = *b* = 104.27, *c* = 88.44	*a* = *b* = 104.44, *c* = 89.64
	*α* = *β* = *γ* = 90°	*α* = *β* = *γ* = 90°
*R*_factor_	0.23	0.24
*R*_free_	0.26	0.27
(%)Favored region	93.8	95.51
(%)Allowed region	3.66	3.09
PDB code	6Y6G	6Y6I

### Structure determination

The structure of *S. typhimurium* WaaB was determined by the single-wavelength anomalous dispersion (SAD) using the iodine derivatization data and CRANK suite (Bailey, 1994). The high resolution structure of *S. typhimurium* apo WaaB and UDP-WaaB structure were determined by molecular replacement and Phaser MR using the *S. typhimurium* WaaB structure as a search model (McCoy et al., 2007). The structures were manually built with Coot (Emsley and Cowtan, 2004), refined with REFMAC5 (Murshudov et al., 2011), and validated by Molprobity (Williams et al., 2018).

### Site-direct mutagenesis and WaaB mutants

The pLOU3-*waaB* plasmid was used as the template for mutagenesis which was performed based on the protocol of Liu and Naismith (2008), a modified version of QuikChange^™^ Site-Directed Mutagenesis System. All mutations were confirmed by DNA sequencing. The mutant plasmids were transformed into the BL21 (DE3). The expression and purification of WaaB mutant proteins were performed using the same protocol as that of the wild-type WaaB.

### WaaB hydrolytic activity determination

All wild-type WaaB and WaaB mutant-MBP fusion proteins were expressed and purified using the method previously described for the expression and purification of the wild-type protein for crystallography.

The hydrolytic activity of WaaB was measured using the UDP-Glo^®^ Glycosyltransferase Assay Kit (Promega) according to the manufacturer’s instructions. A concentration of 100 μM UDP-galactose was found to provide the clearest signal. Wild type and mutant proteins were assayed in a 1 in 2 serial dilution from 200 to 0.775 ng WaaB per well (*n* = 4), along with negative controls (*n* = 3) for UDP-galactose, UDP-Glo^®^ (luciferase), WaaB and empty wells. Measurements were performed in white polystyrene 384-well plates using a HIDEX Sense instrument in top-reading mode with a 1 s counting time at room temperature following a 60 min reaction and 60 min post-reaction incubation. Calculate the average luminescence of four replicate experiments, with concentration as the horizontal axis and average luminescence as the vertical axis, and make a line graph. Student’s *t*-test of the raw data revealed that WaaB concentrations greater than 6.25 ng/well were significantly different (*p* < 0.05) from WaaB negative controls and results at lower concentrations were excluded when comparing activity. In order to determine the overall activity of the mutant compared to the wild type, the relative activity of the mutant at concentrations of 6.25–200 ng/well was calculated using the activity of the wild type at each concentration as 100%, and finally the mean of the relative activity was taken to plot a bar graph.

## Results

### The galactosyltransferase WaaB adopts a GT-B fold

**Figure 2:**
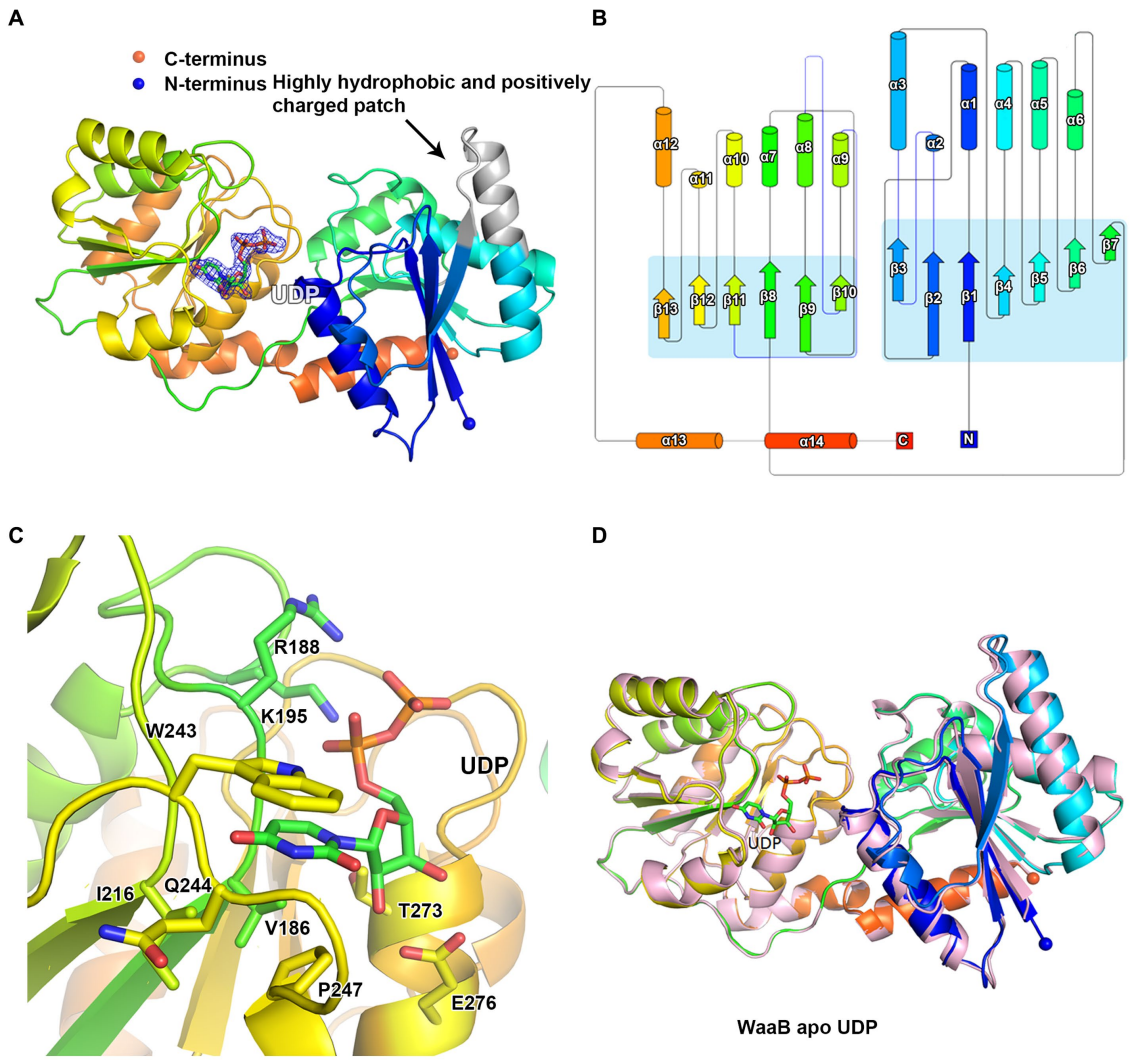
Structure of WaaB. **(A)** Cartoon representation of WaaB. WaaB have N-terminal and C-terminal domains. UDP is bound at the C-terminal domain (donor substrate binding domain). The highly hydrophobic and positively charged patch in grey may be the membrane anchor of WaaB. **(B)** Topology of WaaB. **(C)** WaaB residues involved in UDP binding. **(D)** Superimposition of structures of the apo WaaB and WaaB in complex with UDP.

WaaB was expressed in the pLOU3 vector as a fusion protein with MBP. After TEV protease cleavage of WaaB from the MBP tag, the wild-type WaaB was purified from the tag. WaaB was crystallized and the resulting crystals belong to space group *P*4_1_2_1_2. The structure was determined to 1.8 Å using single-wavelength anomalous dispersion (SAD) data collected at the wavelength 1.80 Å and using the iodine-derived crystals. The overall crystal structure of WaaB is folded into two “Rossmann-like” (*β*/*α*/*β*) domains (Figures 2A,B), the N-terminal domain (the acceptor substrate binding domain) and the C-terminal domain (the donor substrate binding domain), which is a typical GT-B fold. The N-terminal domain comprises residues Met1-Val161 and displays seven parallel β-strands sandwiched by six α-helices with two helices at one side and four helices at the other side. The C-terminal domain contains residues Ala181-Gln358, which consists of six parallel β-strands forming a β-sheet, six α-helices with three of them at one side and the remaining three at the other side of the β-sheet, and an additional two C-terminal α-helices. The first C-terminal α-helix associates with the C-terminal domain, while the last C-terminal helix (residues Asp342-Gln358) joins to the N-terminal domain (Figures 2A,B). Therefore, the N-terminal domain and the C-terminal domain are connected by the C-terminal helices and a long loop consisting of residues Tyr 162 to Pro180 (Figures 2A,B).

A structural homology search using the DALI server identified several GT-B enzyme structures similar to WaaB. TarM, a *Staphylococcus aureus* cell wall teichoic acid alpha-glycosyltransferase (PDB code 4X7M), is similar to WaaB with a *Z*-score of 32.4 and RMSD of 3.0 over 333 aligned residues. HepE, involved in the biosynthesis of *Anabaena heterocyst* envelope polysaccharide (PDB code 4XSO), has a *Z*-score of 31.9 and RMSD of 2.9 over 323 aligned residues. BaBshA, the *Bacillus anthracis* N-acetyl-α-D-glucosaminyl L-malate synthase, has a *Z*-score of 31.7 and RMSD of 3.8 over 331 aligned residues. GtfA/GtfB complex (PDB code 5E9U), a *Streptococcus gordonii* cytodolic O-glycosyltransferase, is a GT-B fold UDP-binding transferase with DALI *Z*-score of 25.1 and RMSD of 3.2 Å over 333 aligned residues, despite sharing only 17% sequence identity with WaaB. These data suggest that despite these GT-B fold glycotransferases being derived from different species and having different functions, they may share similar catalytic mechanisms.

### Donor substrate binding site

To identify the donor substrate UDP-galactose binding site of WaaB, we attempted to soak crystals with UDP-galactose. The structure of WaaB in complex with UDP-galactose was determined to a resolution of 1.9 Å. An additional Fo-Fc electron density appeared in the donor substrate binding domain, the C-terminal domain of WaaB (Figure 2A). This electron density has been identified as a UDP molecule, suggesting that WaaB hydrolyzes the substrate (UDP-galactose) to product (UDP), even in the crystals. This is consistent with other GT-B glycotransferases in which the donor substrates is hydrolyzed by the glycotransferases in the absence of the acceptor substrates (Martinez-Fleites et al., 2006; Albesa-Jove and Guerin, 2016). The UDP molecule binds to the C-terminal domain and is located in the cleft formed by the C-terminal and the N-terminal domains. The uracil of UDP is located in a hydrophobic pocket consisting of residues W243, I216, P247, and V186, where the uracil is anchored by two hydrogen bonds formed between the uracil and the amide and carbonyl groups of Q244. The ribose of UDP is stabilized by two hydrogen bonds with E276 and by hydrophobic interactions with T273, and the two phosphate groups form salt bonds with the side chains of K195 and R188 (Figure 2C).

The overall structure of WaaB in complex with UDP is almost identical to the apo structure of WaaB with an RMSD of 0.5624 Å over 357 Cα atoms, suggesting that binding of the donor product, UDP, does not induce significant conformational changes of the apo WaaB structure (Figure 2D). The N-terminal domain structure showed a minor shift while the C-terminal domain structure remained mostly unchanged to that of the apo state. The helix α5 was slightly stretched into a loop and α2 turned from a loop into a helix after UDP entered the WaaB structure (Figure 2D). However, the conformations of the side chains of W243, P247, V186, T273, E276, K195, and R188 have changed upon UDP binding (Supplementary Figure S1; Table S1). Amino acid sequence analysis showed that residues W243, P247, V186, Q244, E276, I216, T273, K195, and R188 are highly conserved in WaaB (Supplementary Figure S2), suggesting that these residues play important roles in UDP-galactose binding and hydrolysis.

**Figure 3:**
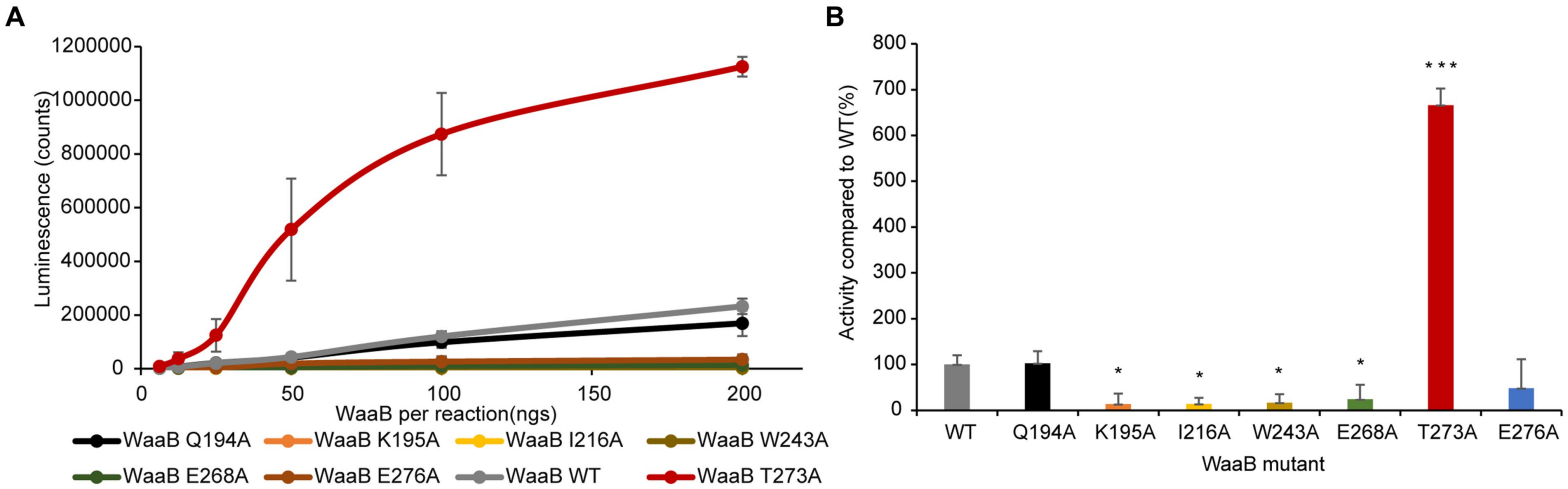
Enzymatic activity of WaaB hydrolysis of UDP-galactose. **(A)** Line diagram of the activity of WaaB and its mutants. **(B)** WaaB mutant activities are represented as the percentages of the wild type. Significant differences from WT activity are denoted by *(*p* < 0.05) and ***(*p* < 0.001), using Student *t*-test.

To test this notion, WaaB mutants K195A, I216A, W243A, T273A, and E276A were generated and the WaaB mutant proteins were overexpressed and purified using the same protocol as for the wild-type WaaB. The galactosyltransferase activities of the WaaB wild type and mutants were determined, which revealed that the activities of mutants K195A, I216A, W243A, and E276A were significantly reduced (Figure 3). Surprisingly, the average activity of the T273A mutant was significantly increased compared with wild-type WaaB. Mutant T273A may produce a more hydrophobic pocket for UDP-galactose binding (Figure 3). These data suggest that these residues are critical for UDP-galactose binding and hydrolysis.

**Figure 4:**
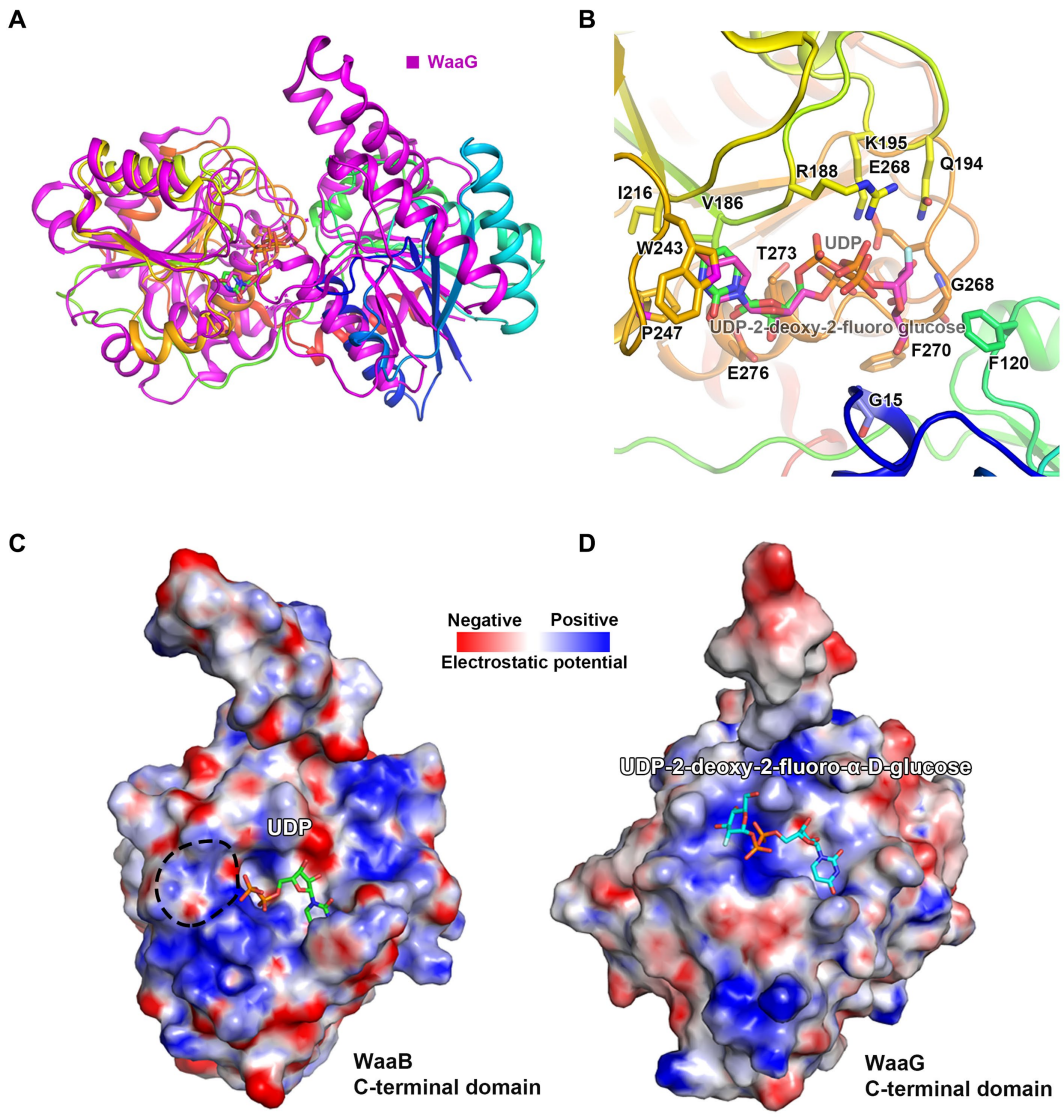
The Donor substrate binding groove of WaaB. **(A)** WaaB and WaaG structures are superimposed over their ligands. **(B)** The potential galactose binding site of WaaB is identified by the structure superimposition. **(C)** The WaaB electrostatic potential map of the potential donor substrate UDP-galactose binding site. **(D)** The WaaG electrostatic potential map of the donor substrate binding site.

In order to identify the residues of WaaB that might bind to the galactose motif of UDP-galactose, we attempted to compare the structures of WaaB in complex with UDP with WaaG in complex with UDP-2-deoxy-2-fluoro glucose. WaaG is a UDP-D-glucosyltransferase directly involved in the biosynthesis of the LPS core oligosaccharide upstream of WaaB. WaaB shares 12.4% amino acid sequence identity with WaaG, and the structure of WaaB is relatively similar to that of WaaG, with an RMSD of 3.2589 Å for 358 aligned calcium atoms (Figure 4A). By comparing the structures of WaaG in complex with UDP-2-fluoro-Glc and WaaB in complex with UDP, we may identify the possible UDP-glycosyl hydrolysis center, as well as the different residues required for the binding of the different substrates. Superimposition of WaaG and WaaB by overlapping their own ligands (Lignalign Pymol), allowed us to identify conserved spatial arrangements local to the UDP-sugars. We found a high degree of similarity between the C-terminal domains of WaaB and WaaG structures, which are mainly responsible for UDP-glycosyl binding (Figure 4A). Similar to WaaB, the uracil of the UDP motif is located in a hydrophobic pocket formed by hydrophobic residues F13, V202, V264, and V234 of WaaG, whereas the ribose motif of the UDP interacts with E289 and R173, as well as V286 (Supplementary Figure S3). The diphosphate forms salt bridges with K209 and R208. However, F13 of WaaG is derived from the N-terminal domain, and UDP-glucose binding requires both the N-terminal and the C-terminal residues of WaaG. This is different from that of the WaaB, and the superimposition of the two structures showed that a rotation of the N-terminal domain of WaaG toward a closing conformation of WaaG (Figure 4A). The glucose motif is located in a pocket formed by E281, A99, D19, A282, A283 and R208 (Supplementary Figure S3). WaaB also forms a pocket at the same position but is formed by E268, G269, F120, F270, G15, and Q194, which may be responsible for galactose binding (Figure 4B). These differences between WaaB and WaaG for the donor substrate binding sites may be important for the selectivity of the donor substrates. To test whether this pocket is important, the potential galactose binding site residues E268 and Q194 were selected for mutagenesis, where E268 in WaaB is conserved and Q194 in WaaB is highly conserved. Galactosyltransferase assays showed that the activity of mutant E268A was significantly reduced (Figure 3), whereas the activity of mutant Q194A was the same as that of the wild type WaaB, indicating that the E268 is critical for the activity of WaaB.

### Putative acceptor substrate binding site

**Figure 5:**
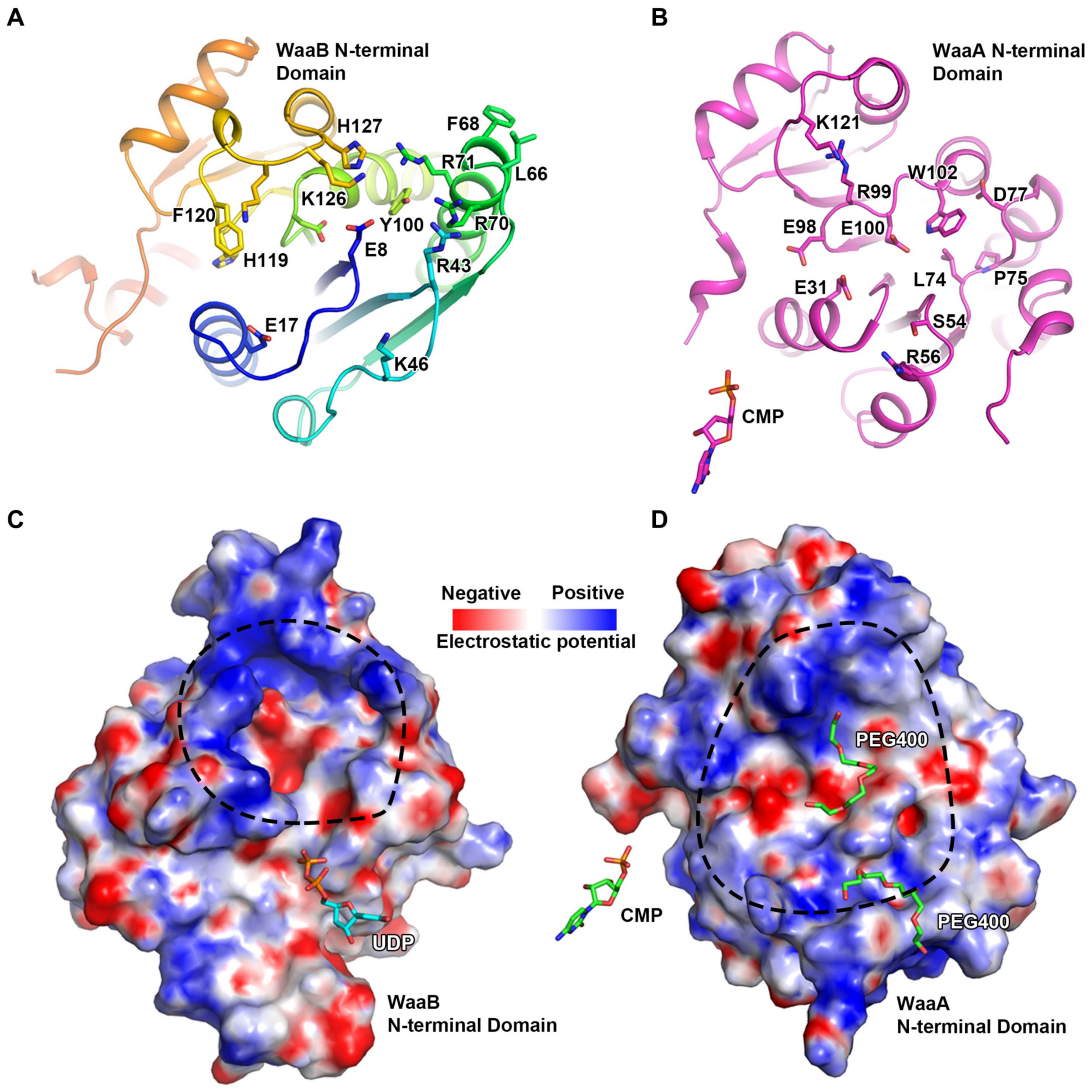
The potential acceptor substrate binding groove of WaaB. **(A)** WaaB potential acceptor substrate binding groove is highly positively charged. **(B)** WaaA acceptor substrate binding groove. **(C)** The electrostatic potential map of the potential WaaB acceptor substrate binding grove. **(D)** The electrostatic potential map of WaaA acceptor substrate binding groove.

Previous studies have demonstrated that the GT-B fold enzyme possessed donor and acceptor binding subsites (Moremen and Haltiwanger, 2019). WaaA, WaaB, WaaG, and WaaL are all involved in the biosynthesis of LPS, the structures of WaaA, WaaG, and WaaL have been resolved (Martinez-Fleites et al., 2006; Schmidt et al., 2012; Ashraf et al., 2022). In the paper of Ashraf et al., they identified two major cavities on the periplasmic side. They hypothesized that the second cavity could be a possible binding site for lipid A of WaaL by MD simulations. In WaaG and WaaA, they found similar grooves and both were hypothesized as receptor binding sites (Martinez-Fleites et al., 2006; Schmidt et al., 2012). In the paper of Schmidt et al., they observed a groove, which they hypothesized as a possible receptor-substrate binding site. Firstly, they analyzed this site by residues and found that E31, S54, L74, P75, D77, E98, E100, and W102 are highly conserved in Kdo transferase. Secondly, they detected a |Fo|-|Fc| density difference at this possible site. Thirdly, by structural analysis of the GT-B enzyme UGT72B1 and other enzymes, they found that the receptor substrates or analogs bind at similar places. Therefore, they hypothesized that the groove observed in the structures is a potential receptor-substrate binding site. Structural analysis of the WaaB shows the di-phosphates of the UDP binding at the donor substrate binding domain point to a groove of the N-terminal domain, the acceptor substrate binding domain. This groove is in close proximity to the UDP. By comparison with the published structure, we speculate that this groove is the putative acceptor substrate binding site for the premature lipopolysaccharide (LPS), Glc-Hep_2_-1-dephospho-Kdo_2_-lipid A (Figure 5A). Indeed, superimposition of WaaB structure in complex with UDP with structures of WaaC, WaaG, and WaaA has revealed that they all have acceptor substrate binding grooves in similar positions (Figure 4A; Supplementary Figures S6, S7). In particular, E17 and G15 of WaaB are absolutely conserved in WaaB homologues and are in the same positions as E31 and G30 of WaaA (Figures 5A,B). The E31 residue is proposed to act as a general base for WaaA, suggesting that E17 of WaaB may be important for the function of WaaB. The potential acceptor substrate binding groove of WaaB is composed of highly conserved residues, E17, H119, F120, K125, K126, H127, E8, D94, R43, R70, L66, F68, and R71 (Figure 5A; Supplementary Figure S2). This groove is highly charged, which is ideal for binding the acceptor substrate Glc-Hep_2_-1-dephospho-Kdo_2_-lipid A (Figure 5A). The potential acceptor substrate binding groove of WaaA is formed by residues E31, S54, L74, P75, D77, E98, E100, W102, R56, R99, and K121 (Figure 5B). Although the two grooves share similarity, they are different in term of electrostatic potential (Figures 5C,D). These differences may be related to the binding of different acceptor substrates. Indeed, no product was obtained when Hep_2_-1-dephospho-Kdo_2_-lipid A (WaaG acceptor substrate) was used as the acceptor substrate for WaaB (Qian et al., 2014).

### Structural basis of the mechanism of WaaB

The donor substrate binding site for WaaB binding to UDP is similar to the UDP binding site of WaaG, consisting of hydrophobic residues W243, V186, and P247 alongside charged residues E276, K195, and R188 (Figure 2C). The potential galactose binding site of WaaB is different from the glucose binding site of WaaG, formed by E268, G269, F120, F270, G15, and Q194 (Figure 4B). The conserved residues E268 and K195 may be the catalytic residues involved in the cleavage of galactose from UDP-galactose. This is consistent with the assay that E268A and K195A mutants significantly reduced UDP-galactose hydrolyzing activity (Figures 3A,B), to within an error of zero. The residues of the donor substrate binding groove of WaaB are different from those of WaaC, WaaA, and WaaG (Figures 4C,D; Supplementary Figures S4, S5), suggesting that this is due to donor substrate selectivity. The GT-B family of transferases is known to have two conformations, an open conformation and a closed conformation (Schmidt et al., 2012). The apo WaaB structure and WaaB in complex with UDP structure are both in an open conformation. We speculate that the acceptor substrate (Glc-Hep_2_-1-dephospho-Kdo_2_-lipid A) binding to the acceptor substrate binding groove could trigger WaaB’s conformational change from the open to the closed conformation for transferring galactose to the acceptor substrate. The residues E17, H119, F120, K125, K126, H127, E8, D94, R43, R70, L66, F68, and R71 comprise the acceptor substrate binding groove (Figure 5A), in which E17 of WaaB is conserved and at the position of the E31 of WaaA, which has been identified as a general base. WaaA has the residue N273 at the position of E268 of WaaB (Figure 5B), while Y96 of WaaG is at the position of E31 of WaaA. Therefore, WaaB shares similarity to WaaG in the donor sugar binding site (E268), as well as the similarity to the WaaA in the acceptor substrate binding site (E17). WaaB can cleave the galactose from UDP-galactose without the acceptor substrate binding. Additionally, both the apo WaaB structure and WaaB in complex with UDP are in the open conformation, suggesting that the E268 of WaaB acts as the general base. However, residue E17 of WaaB is conserved, suggesting that this residue may also play an important role in the formation of the product through contact with the intermediates. The acceptor substrate binding groove is also different from WaaC and WaaA (Supplementary Figures S9, S10), suggesting the selectivity of the acceptor substrate binding groove.

## Conclusion

GTs are a large family of enzymes responsible for carbohydrate biosynthesis, more than one million GT sequences in the CAZy database have been categorized into 116 families based on amino acid sequence similarity (Zhang et al., 2022). Among these GT sequences, only less than 1% have resolved three-dimensional structures. Notably, data from the CAZy database show that only 37 three-dimensional structures have been resolved in the GT4 family. Understanding the relationship between the sequence, three-dimensional structures, and substrate specificity of GTs is a fundamental challenge in glycobiology. WaaB is a GT-B family galactosyltransferase that transfers a galactosyl residue to the outer core of LPS during biosynthesis. First of all, in this paper, we report the first crystal structures of *S. typhimurium* WaaB in the unbound state and in complex with UDP at the resolution of 1.81 Å and 1.90 Å, respectively. Secondly, we confirm that the overall crystal structure of WaaB folds into two “Rossmann-like” (*β*/*α*/*β*) structural domains, the C-terminal and N-terminal structural domains, which are topologically similar to the GTs of the previously described GT-B folded structure. In the UDP complex structure, the UDP molecule binds to the C-terminal structural domain of WaaB. Thirdly, Soaking WaaB crystals with UDP or UDP-galactose produced structures containing UDP but without the galactose electron density, strongly suggesting that the crystal-form WaaB retains the catalytic ability to hydrolyze UDP-galactose to UDP. This suggests that WaaB has the ability to complex UDP but not galactose. Finally, based on the UDP complex structure and superimposition with the related glycosyltransferase WaaG, we propose the UDP-galactose hydrolysis center of WaaB. Mutagenesis and enzyme activity assays focusing on the predicted functional center of WaaB show that K195A, I216A, W243A, E268A, and E276A mutant proteins have reduced activity. These data together with our structures reveal that the hydrophobic pocket next to the UDP binding site is a UDP-galactose hydrolysis center, and we propose K195 and E268 are directly involved in UDP-galactose hydrolysis.

## Discussion

The bilayer membrane system of Gram-negative bacteria provides them with additional protection and enhanced virulence. The asymmetric outer membrane is particularly important as it provides a unique permeability barrier for Gram-negative bacteria (Masi et al., 2017; Sun et al., 2022). The asymmetry of the OM originates from differences in the composition of the inner and the outer leaflets, the inner leaflet is composed of phospholipids while the outer leaflet is made up of a large amphipathic lipopolysaccharide (LPS) (Silhavy et al., 2010; Tang et al., 2021). LPS, distinct from other membrane components, consists of three different moieties: lipid A, core oligosaccharide, and O-antigen (Silhavy et al., 2010; Garcia-Vello et al., 2022). Some of the sugar residues on LPS are essential for the survival of Gram-negative bacterial cells, such as Hep and the two early Kdo in the inner core region. Other sugar residues in the core oligosaccharide region also have various special functions that contribute to increased resistance to different kinds of toxic molecules and enhanced invasion of the host. In the glycosyltransferases (GTs) of LPS biosynthesis, WaaB transfers galactose from UDP-galactose to the intermediate rough LPS. This has the effect of enhancing the resistance to detergents and bile of *S. typhimurium* (King et al., 2009; Su et al., 2009; Qian et al., 2014). Furthermore, it also revealed that this galactose is critical for the invasion of *S. typhimurium*. This suggests WaaB is a potential target for drug discovery.

According to our sequence alignment analysis result, WaaB protein is highly conserved among *E. coli* and *S. typhimurium*. It has been reported that the *E. coli* K-12 and *S. typhimurium* WaaB proteins are functionally equivalent (Pradel et al., 1992). Here we report the first crystal structure of WaaB, which suggests that WaaB shares features with other GT-4 family GTs in adopting a “Rossmann-like” (*β*/*α*/*β*) GT-B folded structure consisting of the C-terminal (the donor substrate binding domain) and N-terminal (the acceptor substrate binding domain) Rossman-like domains. The structure of WaaB in complex with UDP and superimposition with the structure of WaaG in complex with UDP-2-deoxy-2-fluoro glucose revealed the donor substrate binding residues. Single residue mutants Q194A, K195A, I216A, W243A, E268A, T273A, and E276A were generated to determine their enzymatic activities. The galactosyltransferase assay results indicate that residues W243, I216, and E276 are critical for the binding of donor substrate UDP-galactose and that E268 and K195 are involved in the hydrolysis of UDP-galactose, while residue Q194 does not contribute to this activity. Notably, the T273A mutant greatly increased the hydrolytic activity of WaaB on UDP-galactose. The acceptor substrate binding groove was identified by superimposition of WaaB structure with WaaA, WaaC, and WaaG structures, which is consistent with the acceptor substrate binding sites for GftA, GftD, and TarM (Supplementary Figures S11–S13). The sequence identity of the donor substrate binding groove and the acceptor substrate binding groove of WaaB is as low as 10% to WaaA, WaaC, WaaG, and other GT-B family transferases. Nevertheless, the structure of WaaB shows similarity to the GT-B family transferases, whereas the structures of the WaaB donor substrate binding groove and acceptor substrate binding groove are different from those of WaaA, WaaC, and WaaG, which reveals substrate selectivity.

It has been suggested that a patch of the N-terminal domain of WaaA and WaaG, composed of hydrophobic and positively charged residues, is surface exposed and identified as the membrane anchor for accessing their acceptor substrates (Schmidt et al., 2012; Liebau et al., 2015). The WaaB N-terminal domain has a similar patch at a similar position (F61–Y76) (Figure 2A), which may be a putative membrane-binding anchor. This anchor may facilitate the binding of WaaB to the acceptor substrate (Glc-Hep_2_-1-dephospho-Kdo_2_-lipid A), on the cytoplasmic side of the inner membrane. The binding of the acceptor substrate may trigger a conformational change of WaaB from the open state to the closed state, where the donor sugar galactose would be transferred to Glc-Hep_2_-1-dephospho-Kdo_2_-lipid A to form the product Gal-Glc-Hep_2_-1-dephospho-Kdo_2_-lipid A.

## Data availability statement

The datasets presented in this study can be found in online repositories. The names of the repository/repositories and accession number(s) can be found in the article/Supplementary material.

## Author contributions

CD and ZZ conceived the project. YC, JG, GA, ZW and ZZ generated the constructs and performed the protein expression, purification, and crystallization. ZZ collected the diffraction data, determined the structures, and did the mutagenesis, mutant protein purification, and enzyme activity assays. YC, CD, GA, and ZZ wrote and revised the manuscript. All authors contributed to the article and approved the submitted version.

## References

[ref1] Albesa-Jove D. Guerin M. E. (2016). The conformational plasticity of glycosyltransferases. Curr. Opin. Struct. Biol. 40, 23–32. doi: 10.1016/j.sbi.2016.07.007, PMID: 27450114

[ref2] Ashraf K. U. Nygaard R. Vickery O. N. Erramilli S. K. Herrera C. M. McConville T. H.. (2022). Structural basis of lipopolysaccharide maturation by the O-antigen ligase. Nature 604:371. doi: 10.1038/s41586-022-04555-x 35388216 PMC9884178

[ref01] Bailey S. (1994). The Ccp4 Suite - Programs for Protein Crystallography. Acta Crystallographica Section D-Biological Crystallography 50, 760–763. doi: 10.1107/s0907444994003112 15299374

[ref3] Clifton L. A. Ciesielski F. Skoda M. W. A. Paracini N. Holt S. A. Lakey J. H. (2016). The effect of lipopolysaccharide Core oligosaccharide size on the electrostatic binding of antimicrobial proteins to models of the gram negative bacterial outer membrane. Langmuir 32, 3485–3494. doi: 10.1021/acs.langmuir.6b00240, PMID: 27003358 PMC4854487

[ref5] Control C. F. D. Prevention (2003). Outbreaks of salmonella serotype enteritidis infection associated with eating shell eggs--United States, 1999-2001. MMWR Morb. Mortal. Wkly Rep. 51:1149. 12553566

[ref6] Crump J. A. Sjolund-Karlsson M. Gordon M. A. Parry C. M. (2015). Epidemiology, clinical presentation, laboratory diagnosis, antimicrobial resistance, and antimicrobial Management of Invasive Salmonella Infections. Clin. Microbiol. Rev. 28, 901–937. doi: 10.1128/CMR.00002-15, PMID: 26180063 PMC4503790

[ref7] Emsley P. Cowtan K. (2004). Coot: model-building tools for molecular graphics. Acta Crystallogr. D Biol. Crystallogr. 60, 2126–2132. doi: 10.1107/S0907444904019158, PMID: 15572765

[ref8] Galan J. E. (2021). Salmonella typhimurium and inflammation: a pathogen-centric affair. Nat. Rev. Microbiol. 19, 716–725. doi: 10.1038/s41579-021-00561-4, PMID: 34012042 PMC9350856

[ref9] Garcia-Vello P. Di Lorenzo F. Zucchetta D. Zamyatina A. De Castro C. Molinaro A. (2022). Lipopolysaccharide lipid a: a promising molecule for new immunity-based therapies and antibiotics. Pharmacol. Ther. 230:107970. doi: 10.1016/j.pharmthera.2021.107970, PMID: 34454000

[ref10] Hoare A. Bittner M. Carter J. Alvarez S. Zaldivar M. Bravo D.. (2006). The outer core lipopolysaccharide of Salmonella enterica serovar typhi is required for bacterial entry into epithelial cells. Infect. Immun. 74, 1555–1564. doi: 10.1128/IAI.74.3.1555-1564.2006 16495526 PMC1418631

[ref02] Kabsch W. (2010). Xds. Salmonella typhimurium and inflammation: a pathogen-centric affair. 66, 125–132. doi: 10.1107/S0907444909047337, PMID: 34012042 PMC9350856

[ref11] King J. D. Erin D. K. N. Westman L. Lam J. S. (2009). Lipopolysaccharide biosynthesis in Pseudomonas aeruginosa. Innate Immun. 15, 261–312. doi: 10.1177/1753425909106436, PMID: 19710102

[ref12] Liebau J. Pettersson P. Szpryngiel S. Maler L. (2015). Membrane interaction of the glycosyltransferase WaaG. Biophys. J. 109, 552–563. doi: 10.1016/j.bpj.2015.06.036, PMID: 26244737 PMC4571021

[ref13] Liu H. Naismith J. H. (2008). An efficient one-step site-directed deletion, insertion, single and multiple-site plasmid mutagenesis protocol. BMC Biotechnol. 8:91. doi: 10.1186/1472-6750-8-91, PMID: 19055817 PMC2629768

[ref14] Martinez-Fleites C. Proctor M. Roberts S. Bolam D. N. Gilbert H. J. Davies G. J. (2006). Insights into the synthesis of lipopolysaccharide and antibiotics through the structures of two retaining glycosyltransferases from family GT4. Chem. Biol. 13, 1143–1152. doi: 10.1016/j.chembiol.2006.09.005 17113996

[ref15] Masi M. Refregiers M. Pos K. M. Pages J. M. (2017). Mechanisms of envelope permeability and antibiotic influx and efflux in gram-negative bacteria. Nat. Microbiol. 2:17001. doi: 10.1038/nmicrobiol.2017.1 28224989

[ref16] McCoy A. J. Grosse-Kunstleve R. W. Adams P. D. Winn M. D. Storoni L. C. Read R. J. (2007). Phaser crystallographic software. J. Appl. Crystallogr. 40, 658–674. doi: 10.1107/S0021889807021206, PMID: 19461840 PMC2483472

[ref17] Moremen K. W. Haltiwanger R. S. (2019). Emerging structural insights into glycosyltransferase-mediated synthesis of glycans. Nat. Chem. Biol. 15, 853–864. doi: 10.1038/s41589-019-0350-2, PMID: 31427814 PMC6820136

[ref18] Morgan E. Campbell J. D. Rowe S. C. Bispham J. Stevens M. P. Bowen A. J.. (2004). Identification of host-specific colonization factors of Salmonella enterica serovar typhimurium. Mol. Microbiol. 54, 994–1010. doi: 10.1111/j.1365-2958.2004.04323.x, PMID: 15522082

[ref19] Murshudov G. N. Skubak P. Lebedev A. A. Pannu N. S. Steiner R. A. Nicholls R. A.. (2011). REFMAC5 for the refinement of macromolecular crystal structures. Acta Crystal. D 67, 355–367. doi: 10.1107/S0907444911001314, PMID: 21460454 PMC3069751

[ref20] Okuda S. Sherman D. J. Silhavy T. J. Ruiz N. Kahne D. (2016). Lipopolysaccharide transport and assembly at the outer membrane: the PEZ model. Nat. Rev. Microbiol. 14, 337–345. doi: 10.1038/nrmicro.2016.25, PMID: 27026255 PMC4937791

[ref21] Pagnout C. Sohm B. Razafitianamaharavo A. Caillet C. Offroy M. Leduc M.. (2019). Pleiotropic effects of rfa-gene mutations on Escherichia coli envelope properties. Sci. Rep. 9:9696. doi: 10.1038/s41598-019-46100-3, PMID: 31273247 PMC6609704

[ref22] Pradel E. Parker C. T. Schnaitman C. A. (1992). Structures of the Rfab, Rfai, Rfaj, and Rfas genes of Escherichia-Coli K-12 and their roles in assembly of the lipopolysaccharide Core. J. Bacteriol. 174, 4736–4745. doi: 10.1128/jb.174.14.4736-4745.1992, PMID: 1624461 PMC206270

[ref23] Qian J. Garrett T. A. Raetz C. R. (2014). In vitro assembly of the outer core of the lipopolysaccharide from Escherichia coli K-12 and Salmonella typhimurium. Biochemistry 53, 1250–1262. doi: 10.1021/bi4015665, PMID: 24479701 PMC3985525

[ref24] Ruan X. Loyola D. E. Marolda C. L. Perez-Donoso J. M. Valvano M. A. (2012). The WaaL O-antigen lipopolysaccharide ligase has features in common with metal ion-independent inverting glycosyltransferases. Glycobiology 22, 288–299. doi: 10.1093/glycob/cwr150, PMID: 21983211

[ref25] Schmid J. Heider D. Wendel N. J. Sperl N. Sieber V. (2016). Bacterial glycosyltransferases: challenges and opportunities of a highly diverse enzyme class toward tailoring natural products. Front. Microbiol. 7:182. doi: 10.3389/fmicb.2016.00182, PMID: 26925049 PMC4757703

[ref26] Schmidt H. Hansen G. Singh S. Hanuszkiewicz A. Lindner B. Fukase K.. (2012). Structural and mechanistic analysis of the membrane-embedded glycosyltransferase WaaA required for lipopolysaccharide synthesis. Proc. Natl. Acad. Sci. 109, 6253–6258. doi: 10.1073/pnas.1119894109 22474366 PMC3341020

[ref27] Silhavy T. J. Kahne D. Walker S. (2010). The bacterial cell envelope. Cold Spring Harb. Perspect. Biol. 2:a000414. doi: 10.1101/cshperspect.a000414, PMID: 20452953 PMC2857177

[ref28] Simpson B. W. Trent M. S. (2019). Pushing the envelope: LPS modifications and their consequences. Nat. Rev. Microbiol. 17, 403–416. doi: 10.1038/s41579-019-0201-x, PMID: 31142822 PMC6913091

[ref29] Su J. Timbely D. Zhu M. Hua X. Liu B. Pang Y.. (2009). RfaB, a galactosyltransferase, contributes to the resistance to detergent and the virulence of Salmonella enterica serovar enteritidis. Med. Microbiol. Immunol. 198, 185–194. doi: 10.1007/s00430-009-0115-8, PMID: 19404677

[ref30] Sun J. W. Rutherford S. T. Silhavy T. J. Huang K. C. (2022). Physical properties of the bacterial outer membrane. Nat. Rev. Microbiol. 20, 236–248. doi: 10.1038/s41579-021-00638-0, PMID: 34732874 PMC8934262

[ref31] Tang X. D. Chang S. H. Qiao W. Luo Q. H. Chen Y. J. Jia Z. Y.. (2021). Structural insights into outer membrane asymmetry maintenance in gram-negative bacteria by MlaFEDB. Nat. Struct. Mol. Biol. 28:532. doi: 10.1038/s41594-020-00532-y, PMID: 33199922

[ref32] Wang J. L. Ma W. J. Fang Y. Liang H. Yang H. T. Wang Y. W.. (2021). Core oligosaccharide portion of lipopolysaccharide plays important roles in multiple antibiotic resistance in Escherichia coli. Antimicrob. Agents Chemother. 65, e00341–e00321. doi: 10.1128/AAC.00341-21 34310209 PMC8448134

[ref33] Wang X. Y. Quinn P. J. (2010). Lipopolysaccharide: biosynthetic pathway and structure modification. Prog. Lipid Res. 49, 97–107. doi: 10.1016/j.plipres.2009.06.002, PMID: 19815028

[ref34] Williams C. J. Headd J. J. Moriarty N. W. Prisant M. G. Videau L. L. Deis L. N.. (2018). MolProbity: more and better reference data for improved all-atom structure validation. Protein Sci. 27, 293–315. doi: 10.1002/pro.3330, PMID: 29067766 PMC5734394

[ref35] Williams D. M. Ovchinnikova O. G. Koizumi A. Mainprize I. L. Kimber M. S. Lowary T. L.. (2017). Single polysaccharide assembly protein that integrates polymerization, termination, and chain-length quality control. Proc. Natl. Acad. Sci. U. S. A. 114, E1215–E1223. doi: 10.1073/pnas.1613609114, PMID: 28137848 PMC5321029

[ref36] Zhang P. Zhang L. J. Jiang X. K. Diao X. T. Li S. Li D. D.. (2022). Docking-guided rational engineering of a macrolide glycosyltransferase glycodiversifies epothilone B. Commun. Biol. 5:3047. doi: 10.1038/s42003-022-03047-y, PMID: 35087210 PMC8795383

[ref37] Zhang P. Zhang Z. Zhang L. J. Wang J. J. Wu C. S. (2020). Glycosyltransferase GT1 family: phylogenetic distribution, substrates coverage, and representative structural features. Comput. Struct. Biotechnol. J. 18, 1383–1390. doi: 10.1016/j.csbj.2020.06.003, PMID: 32637037 PMC7316871

