# Supplementary material for: Crystal structure of the lipopolysaccharide outer core galactosyltransferase WaaB involved in pathogenic bacterial invasion of host cells
Source: Front Microbiol. 2023 Sep 22;14:1239537. doi: 10.3389/fmicb.2023.1239537 (PMC10556518; doi:10.3389/fmicb.2023.1239537)
Supplement: Supplementary file 1 [file Data_Sheet_1.PDF]

Supplementary Material

Crystal structure of the lipopolysaccharide outer core galactosyltransferase WaaB involved in pathogenic bacterial invasion of host cells

Yatian Chen[1,2], Jiayue Gu[1,2], Gareth Ashworth[3], Zhengyu Zhang1,2*, Changjiang Dong[1,2*]

***Correspondence:**

Corresponding Author: Changjiang Dong, Zhengyu Zhang

changjiangdong@whu.edu.cn, zhengyu.zhang@whu.edu.cn

Supplementary Figures

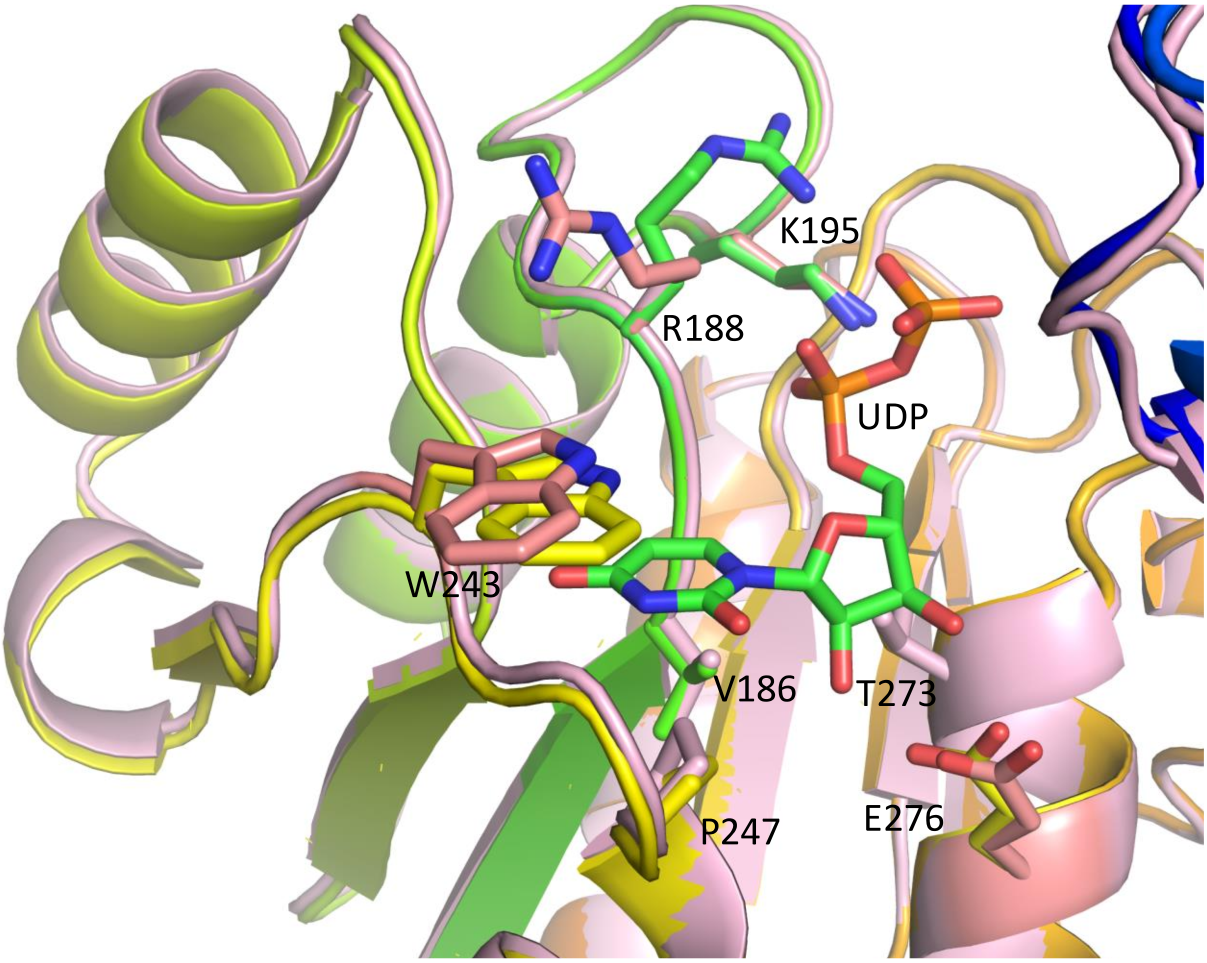

Figure S 1: Conformational changes of donor substrate binding residues upon UDP binding. WaaB Apo protein is in warmpink, and WaaB in complex with UDP is in rainbow.

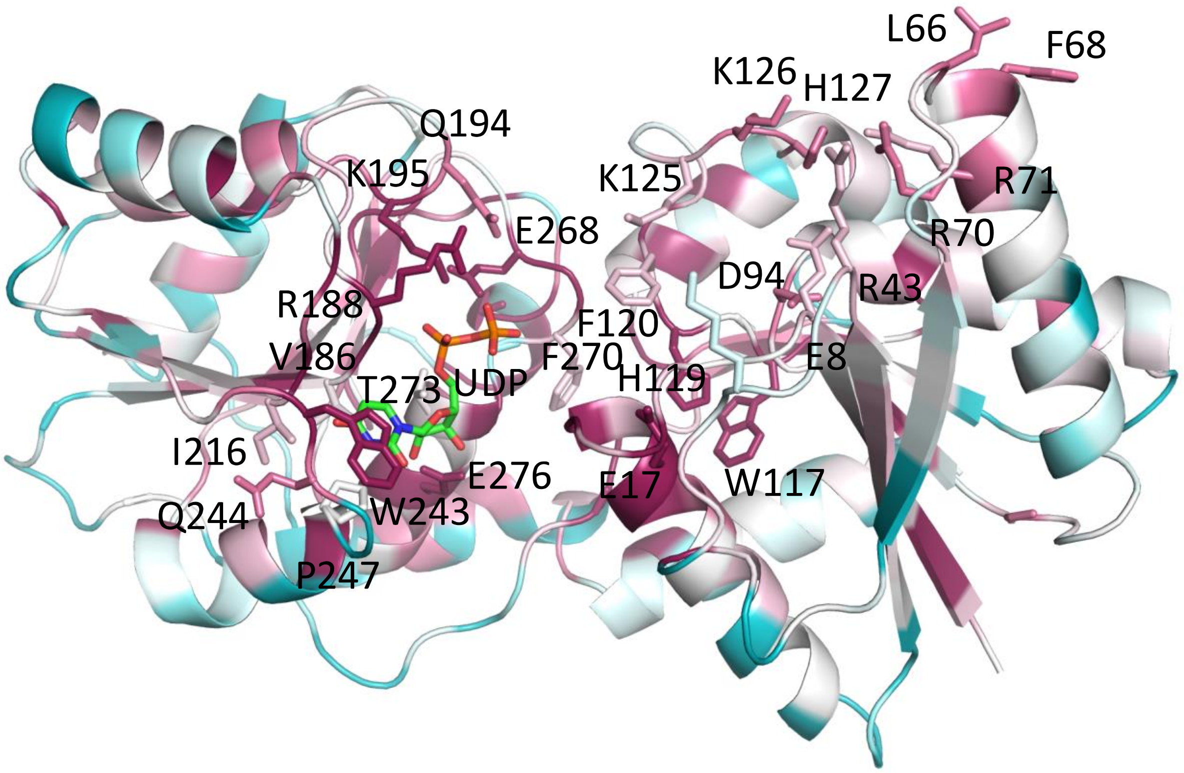

Figgure S 2. Conserved residues of WaaB. The conserved residues of WaaB were analysed using the Consurf server. The most variable residues are in cyan, and the most conserved residues are in dark red.

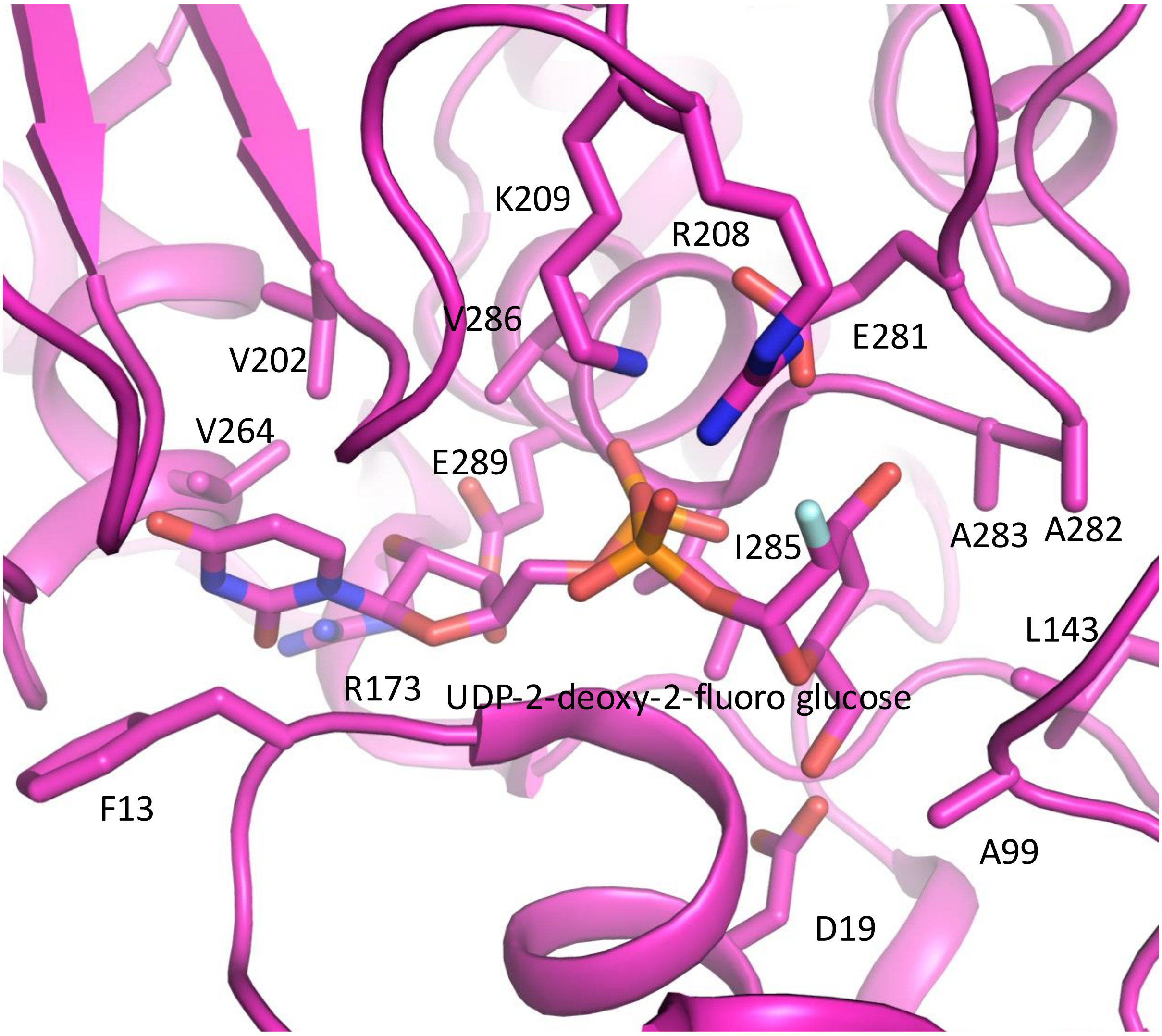

Figure S 3. WaaG donor substrate binding site. The UDP-2-deoxy-2-fluoro glucose is located in the WaaG donor substrate binding site.

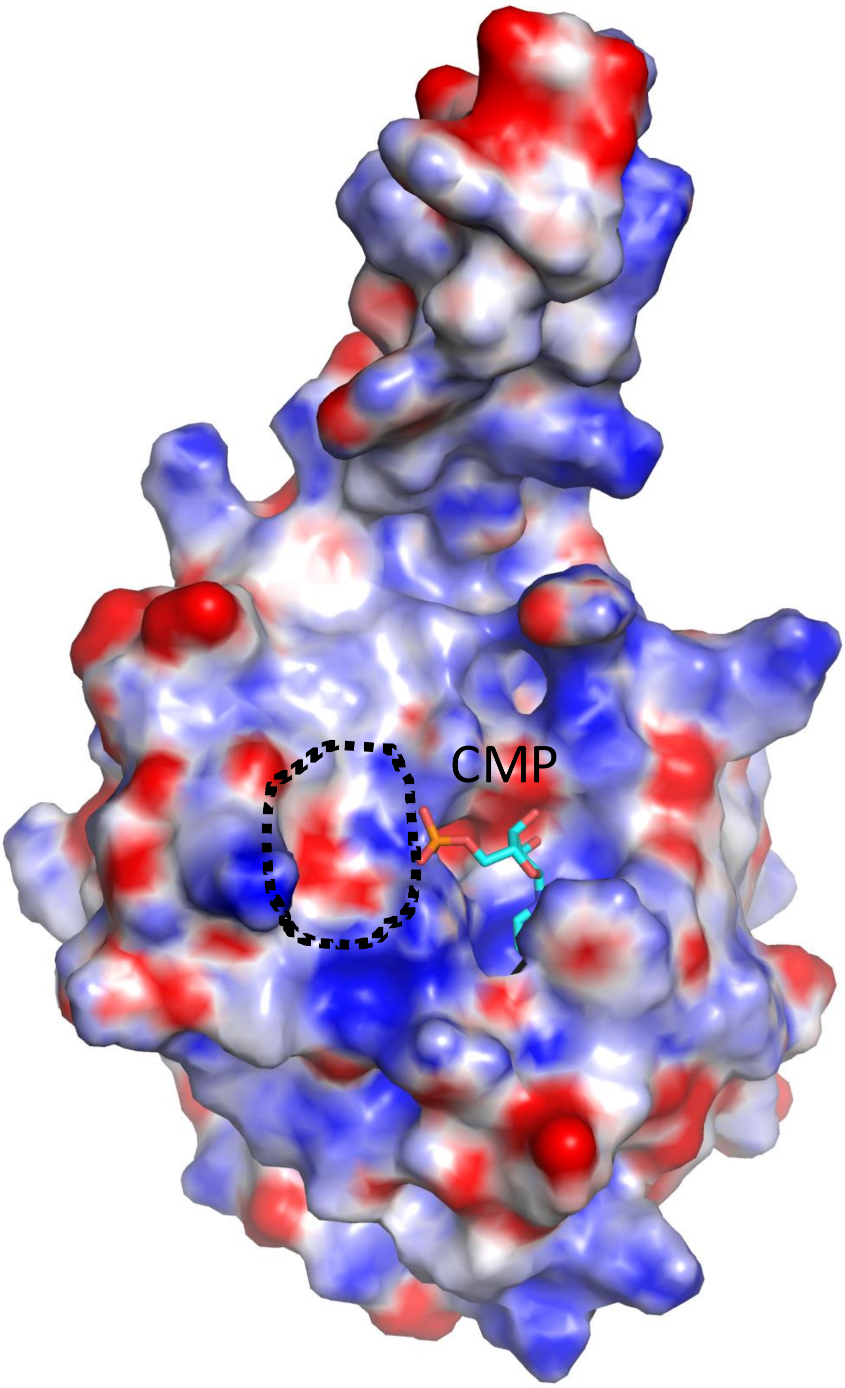

Figure S 4. Electrostatic potential map of WaaA donor substrate binding site. The dotted circle is the potential donor sugar motif binding site.

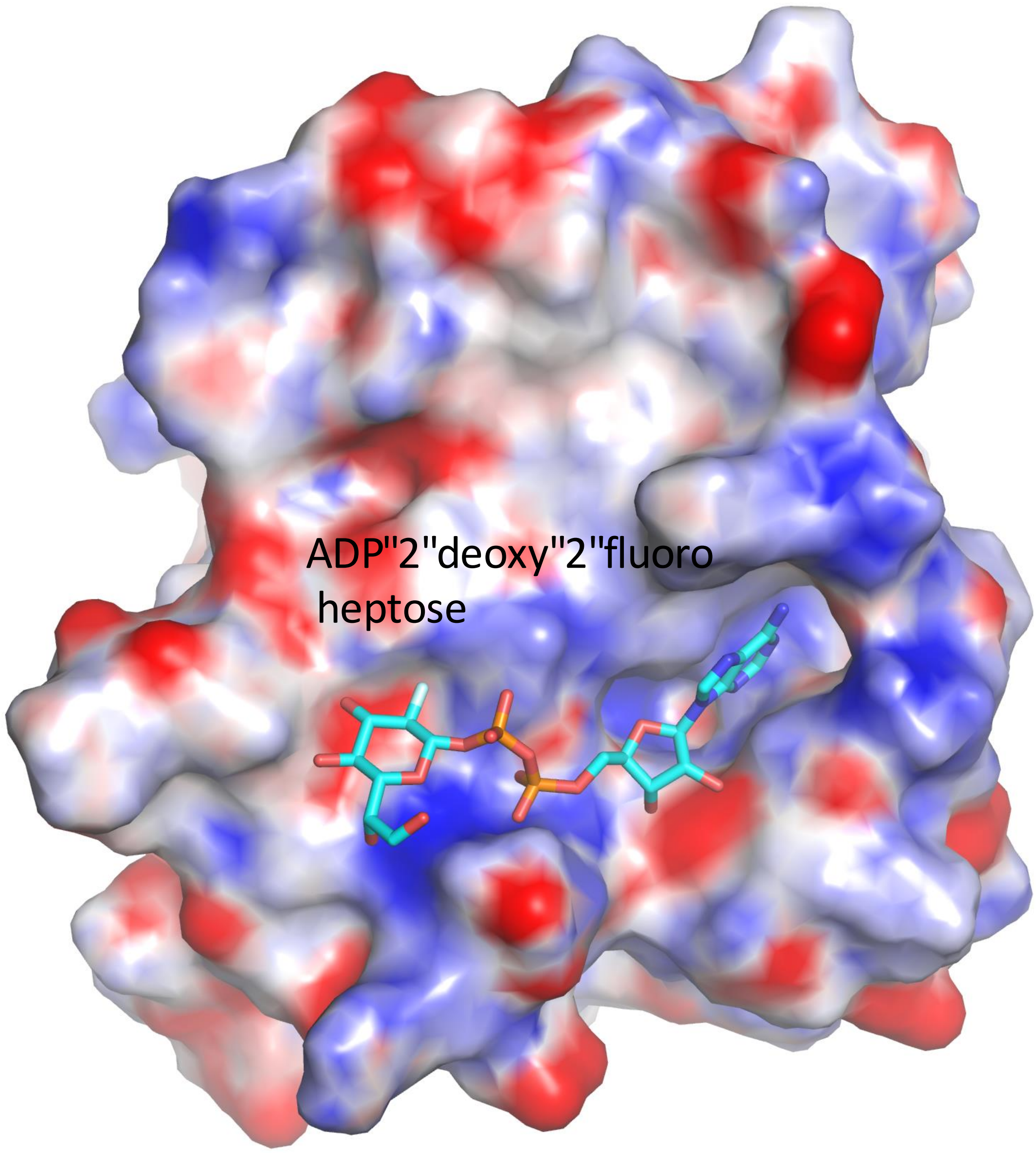

Figure S 5. Electrostatic potential map of WaaC donor substrate binding site. ADP-2-deoxy-2-fluoro-heptose binds at the donor substrate binding site.

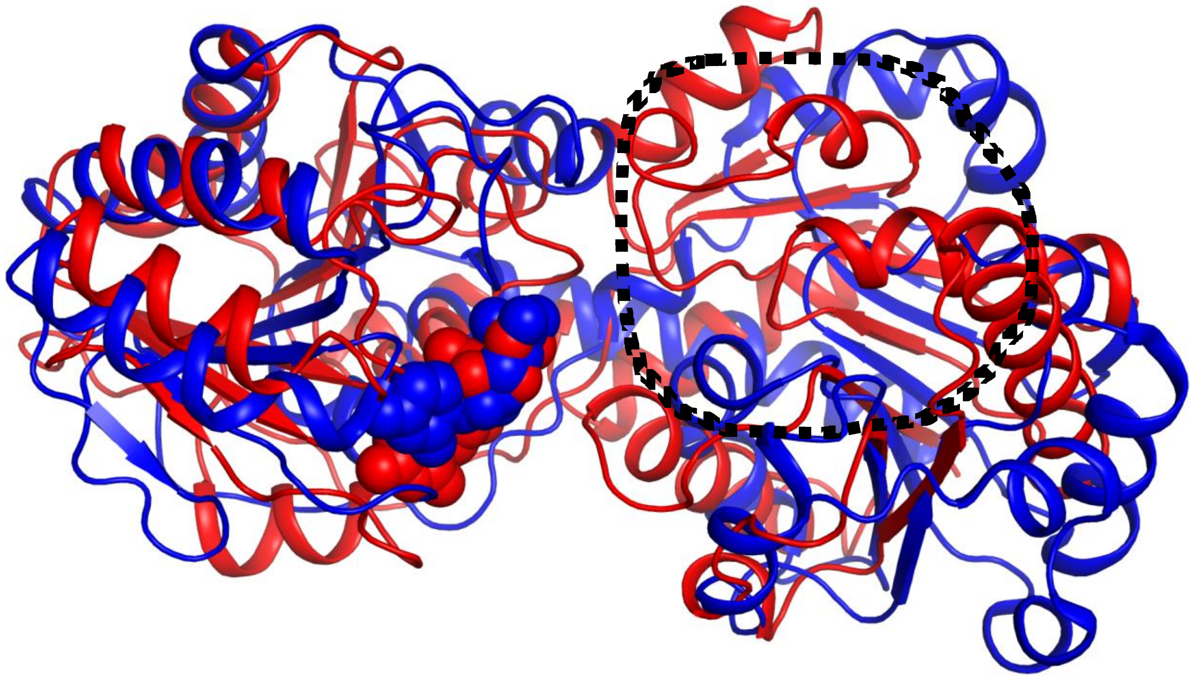

Figure S 6. Structural superimposition of WaaB and WaaA. WaaB is in red and WaaA is in blue. The dotted circle is the acceptor substrate binding groove.

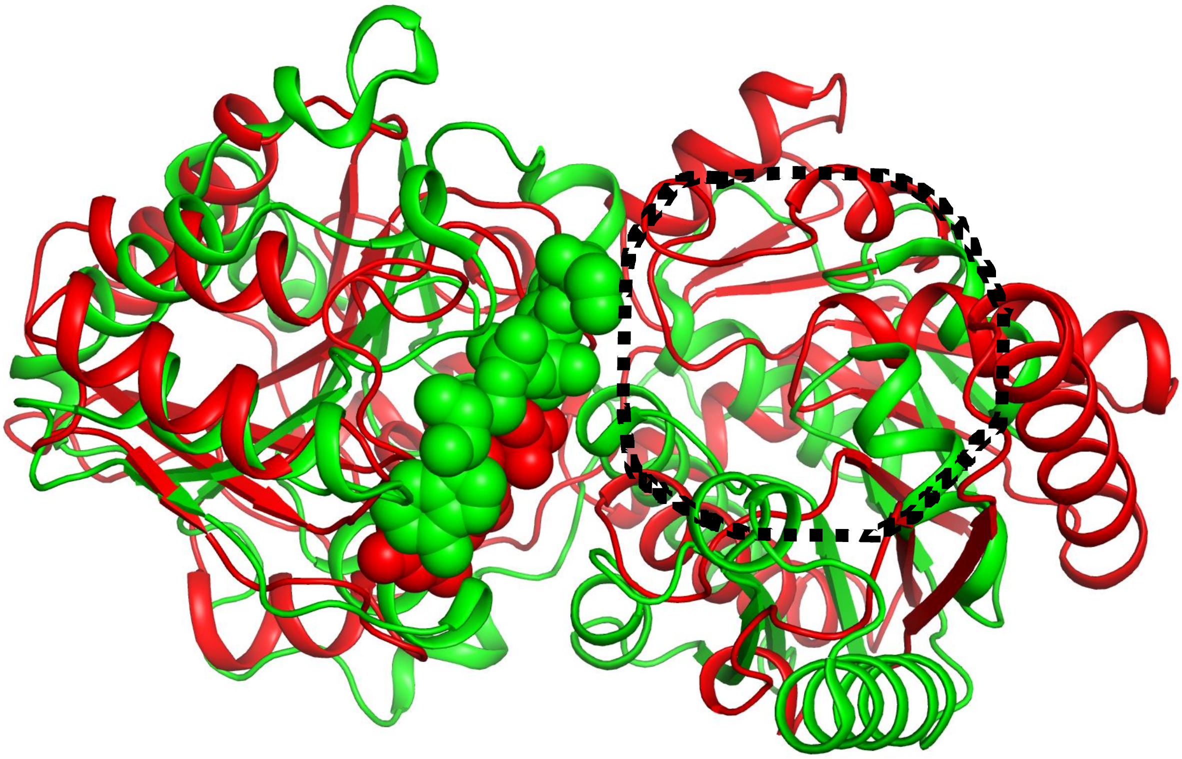

Figure S 7. Structural superimposition of WaaB and WaaC . WaaB is in red and WaaC is in green. The dotted circle is the acceptor substrate binding groove.

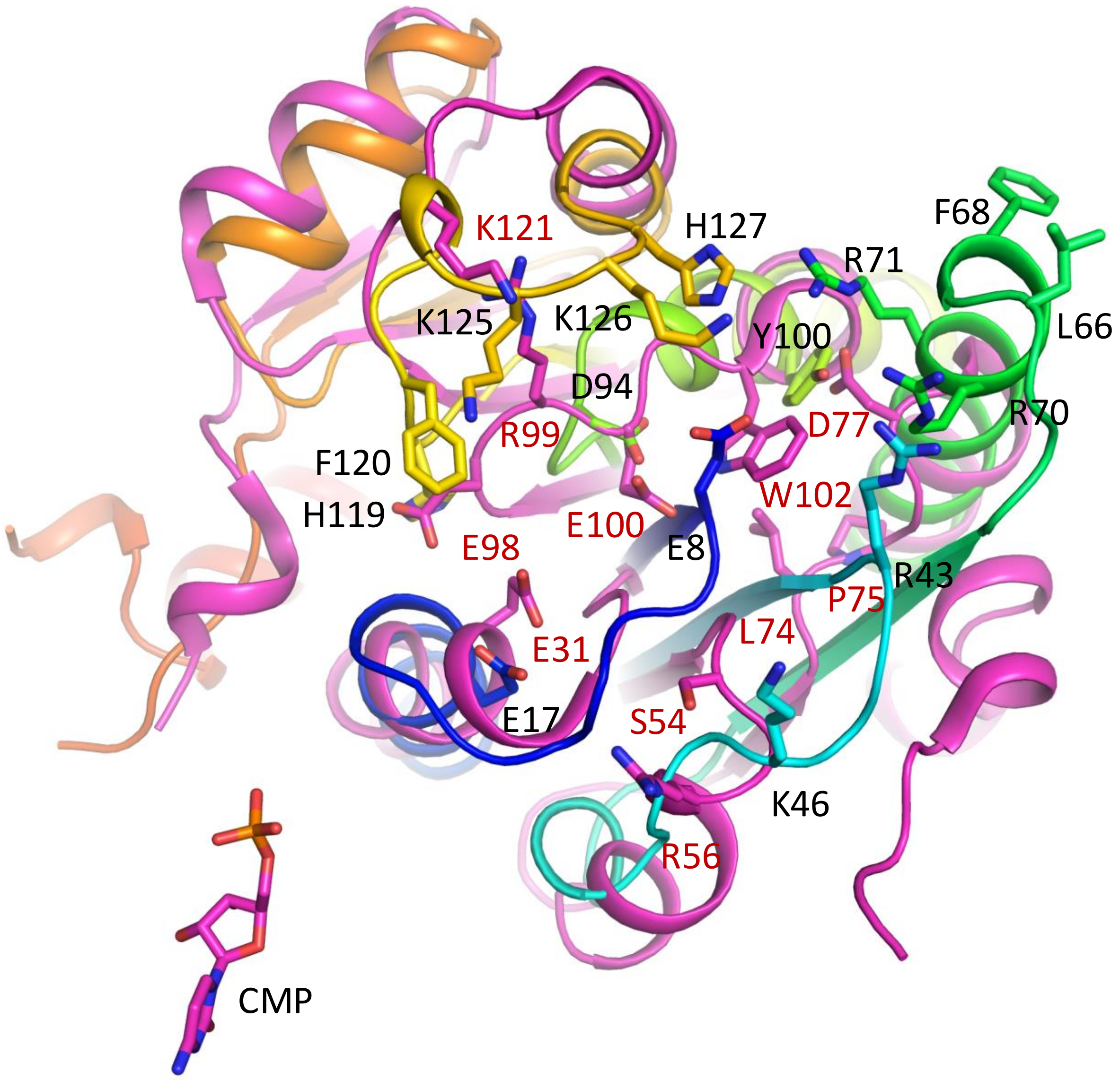

Figure S 8. The superimposition of the acceptor substrate binding sites of WaaB and WaaA. The WaaB is in rainbow and WaaA is in purple.

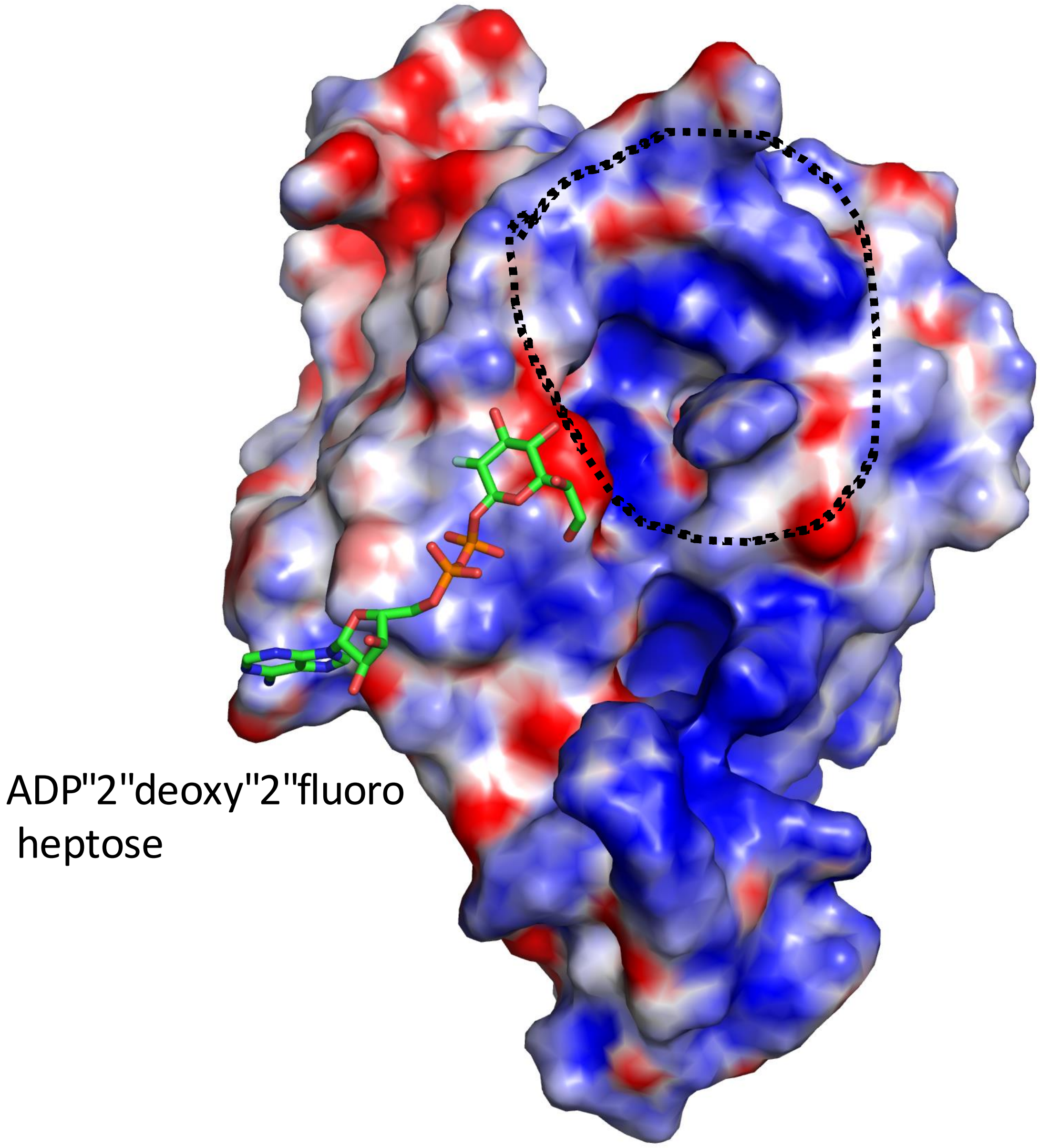

Figure S 9. Electrostatic potential map of WaaC acceptor substrate binding domain. The dotted circle is the acceptor substrate binding site.

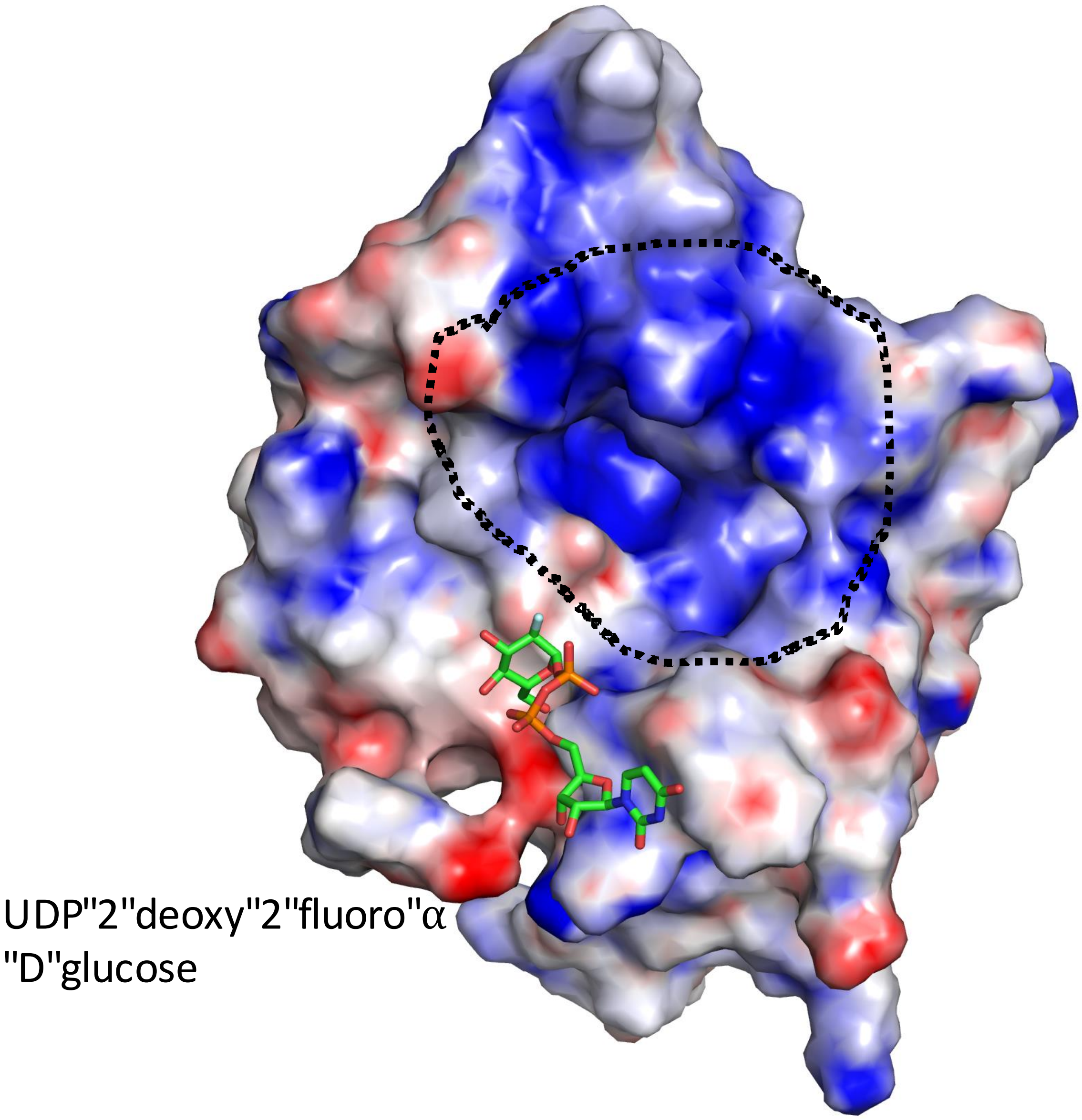

Figure S 10. Electrostatic potential map of WaaG acceptor substrate binding domain. The dotted circle is the acceptor substrate binding site.

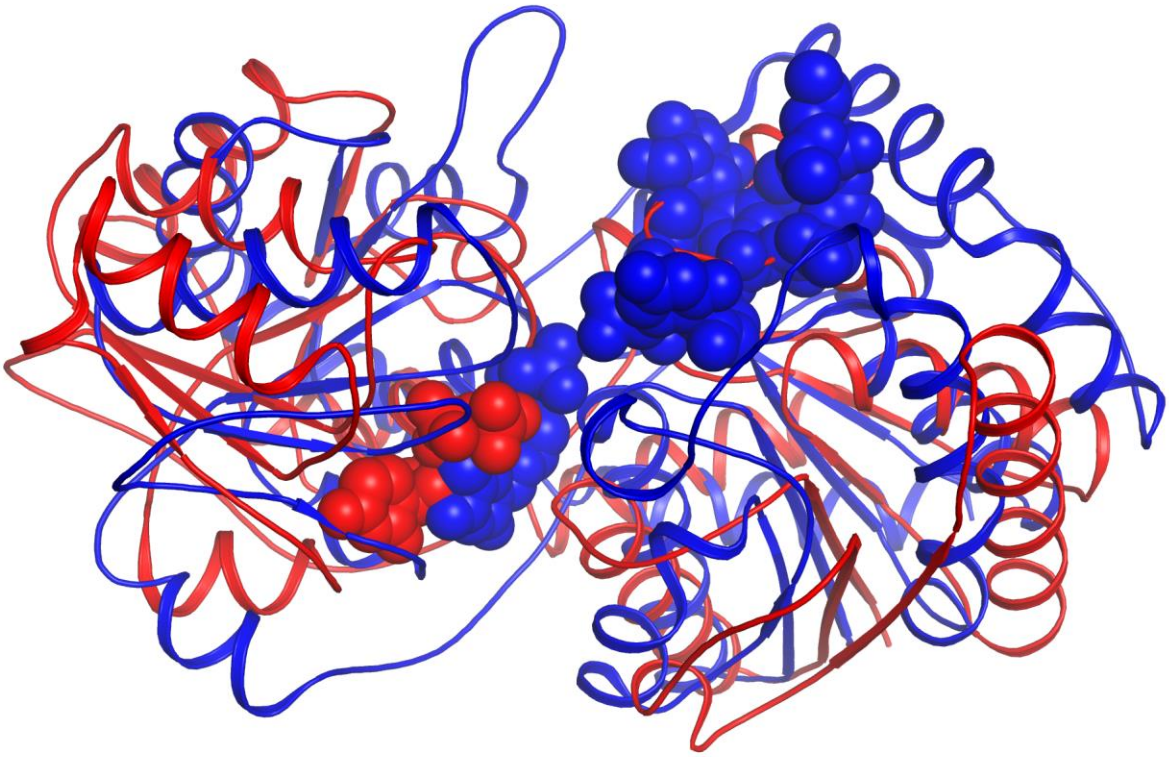

Figure S 11. Superimposition of WaaB and GtfA. WaaB in red, and GtfA in blue. The vamcomycin is bound at the substrate acceptor binding site.

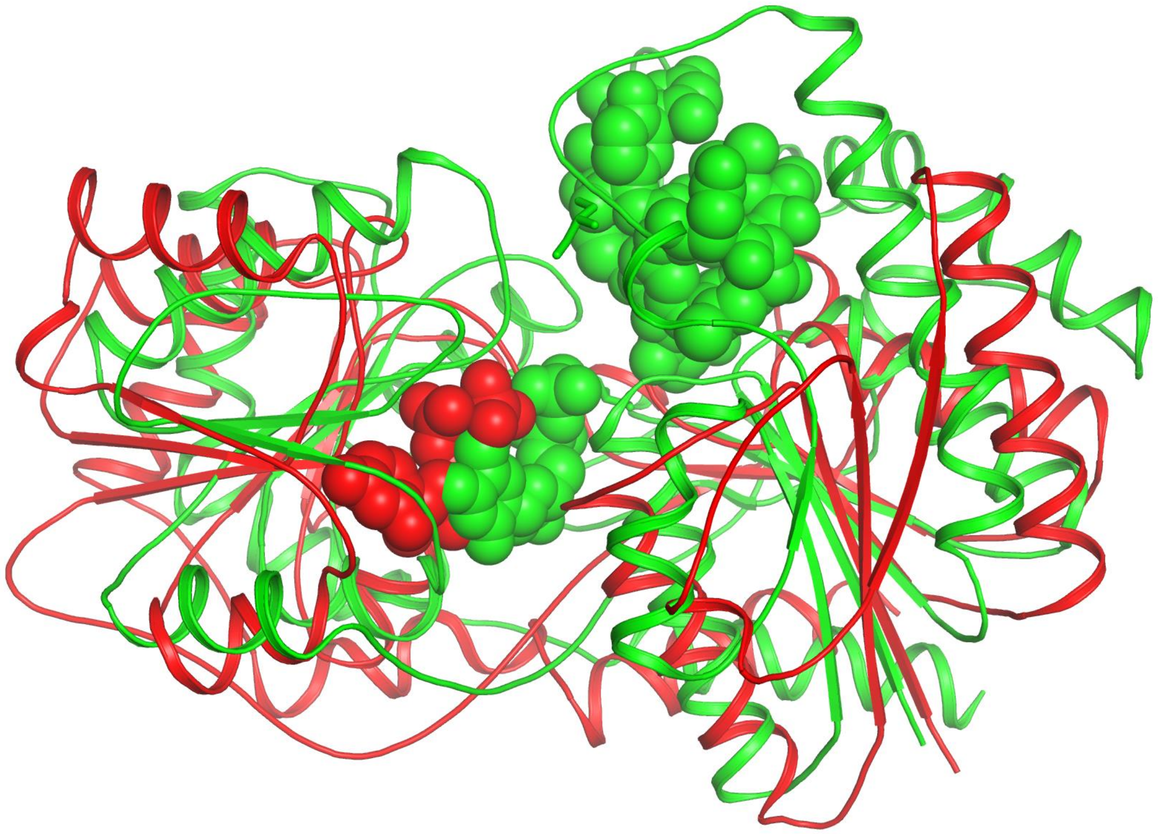

Figure S 12. Superimposition of WaaB and GtfD. WaaB in red, and GtfD in green. The desvancosaminyl vamcomycin is bound at the substrate acceptor binding site.

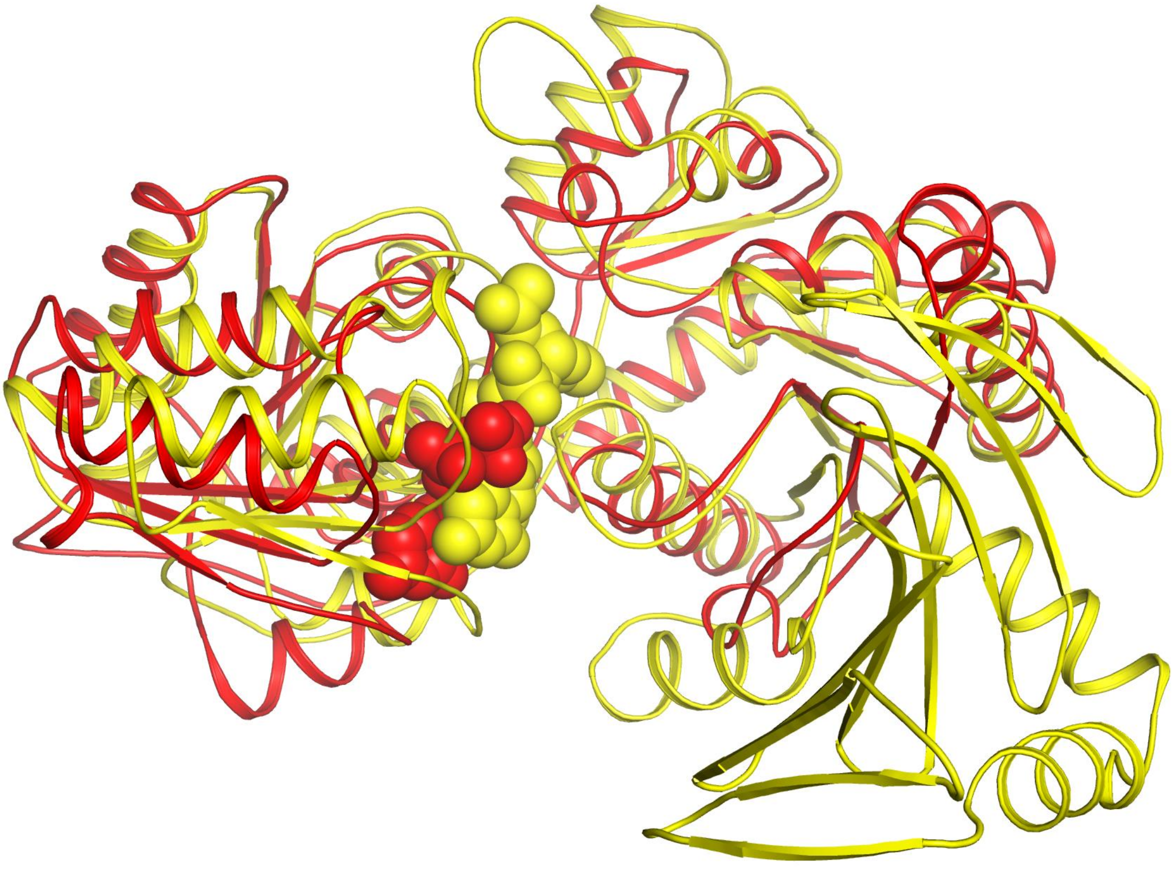

Figure S 13. Superimposition of WaaB and TarM. WaaB in red, and TarM in yellow.
