# Supplementary material for: Crystal structure of the lipopolysaccharide outer core galactosyltransferase WaaB involved in pathogenic bacterial invasion of host cells
Source: Front Microbiol. 2023 Sep 22;14:1239537. doi: 10.3389/fmicb.2023.1239537 (PMC10556518; doi:10.3389/fmicb.2023.1239537)
Supplement: Supplementary file 2 [file Table_1.docx]

Supplementary Table S1 Strains used for homology search in this experiment.

| \| **Organisms** \| **Gram-negative/gram-positive** \| \| --- \| --- \| \| *Achromobacter arsenitoxydans SY8* \| gram-negative \| \| *Achromobacter insuavis AXX-A* \| gram-negative \| \| *Achromobacter marplatensis* \| gram-negative \| \| *Achromobacter piechaudii ATCC 43553* \| gram-negative \| \| *Achromobacter sp. RTa* \| gram-negative \| \| *Achromobacter xylosoxidans (strain A8)* \| gram-negative \| \| *Acinetobacter brisouii CIP 110357* \| gram-negative \| \| *Acinetobacter rudis CIP 110305* \| gram-negative \| \| *Acinetobacter sp. ANC 3789* \| gram-negative \| \| *Acinetobacter sp. CIP 101966* \| gram-negative \| \| *Acinetobacter sp. CIP 102159* \| gram-negative \| \| *Acinetobacter sp. CIP A162* \| gram-negative \| \| *Acinetobacter sp. COS3* \| gram-negative \| \| *Advenella kashmirensis (strain DSM 17095 / LMG 22695 / WT001) (Tetrathiobacter kashmirensis)* \| gram-negative \| \| *Advenella kashmirensis W13003* \| gram-negative \| \| *Advenella mimigardefordensis (strain DSM 17166 / LMG 22922 / DPN7)* \| gram-negative \| \| *Alcaligenes sp. EGD-AK7* \| gram-negative \| \| *Alcaligenes xylosoxydans xylosoxydans (Achromobacter xylosoxidans)* \| gram-negative \| \| *Alistipes sp. CAG:831* \| gram-negative \| \| *Alkalihalobacillus clausii (strain KSM-K16) (Bacillus clausii)* \| gram-positive \| \| *Allocoleopsis franciscana PCC 7113* \| gram-negative \| \| *Ammonifex degensii (strain DSM 10501 / KC4)* \| gram-negative \| \| *Arsenophonus nasoniae (son-killer infecting Nasonia vitripennis)* \| gram-negative \| \| *Bacillus cereus* \| gram-positive \| \| *Bacillus cereus (strain G9842)* \| gram-positive \| \| *Bacillus sp. UMTAT18* \| gram-positive \| \| *Bacillus thuringiensis serovar thuringiensis str. IS5056* \| gram-positive \| \| *Bacillus wiedmannii* \| gram-positive \| \| *bacteria symbiont BFo1 of Frankliniella occidentalis* \| gram-negative \| \| *Bacteroides thetaiotaomicron (strain ATCC 29148 / DSM 2079 / JCM 5827 / CCUG 10774 / NCTC 10582 / VPI-5482 / E50)* \| gram-negative \| \| *Bacteroidetes oral taxon 274 str. F0058* \| gram-negative \| \| *Bordetella avium (strain 197N)* \| gram-negative \| \| *Bordetella parapertussis (strain Bpp5)* \| gram-negative \| \| *Brenneria goodwinii* \| gram-negative \| \| *Burkholderia sp. MSHR3999* \| gram-negative \| \| *Buttiauxella agrestis ATCC 33320* \| gram-negative \| \| *Caballeronia glathei* \| gram-negative \| \| *Caldanaerobacter subterraneus subsp. pacificus DSM 12653* \| gram-positive \| \| *Calothrix sp. PCC 6303* \| gram-negative \| \| *Calothrix sp. PCC 7507* \| gram-negative \| \| *Candidatus Entotheonella factor* \| gram-negative \| \| *Candidatus Entotheonella gemina* \| gram-negative \| \| *Candidatus Paracaedibacter acanthamoebae* \| gram-negative \| \| *Candidatus Wolfebacteria bacterium GW2011_GWE2_44_13* \| gram-negative \| \| *Castellaniella defragrans 65Phen* \| gram-negative \| \| *Cedecea davisae DSM 4568* \| gram-negative \| \| *Chromohalobacter salexigens (strain ATCC BAA-138 / DSM 3043 / CIP 106854 / NCIMB 13768 / 1H11)* \| gram-negative \| \| *Chroococcidiopsis thermalis (strain PCC 7203)* \| gram-negative \| \| *Chryseobacterium koreense CCUG 49689* \| gram-negative \| \| *Citrobacter amalonaticus Y19* \| gram-negative \| \| *Citrobacter rodentium (strain ICC168) (Citrobacter freundii biotype 4280)* \| gram-negative \| \| *Citrobacter sp. KTE151* \| gram-negative \| \| *Coprothermobacter proteolyticus (strain ATCC 35245 / DSM 5265 / OCM 4 / BT)* \| gram-negative \| \| *Croceibacterium atlanticum* \| gram-negative \| \| *Cronobacter malonaticus* \| gram-negative \| \| *Cupriavidus sp. SK-3* \| gram-negative \| \| *Cupriavidus taiwanensis (strain DSM 17343 / BCRC 17206 / CCUG 44338 / CIP 107171 / LMG 19424 / R1) (Ralstonia taiwanensis (strain LMG 19424))* \| gram-negative \| \| *Deinococcus geothermalis (strain DSM 11300 / AG-3a)* \| gram-positive \| \| *Deinococcus phoenicis* \| gram-positive \| \| *Desulfofarcimen acetoxidans (strain ATCC 49208 / DSM 771 / KCTC 5769 / VKM B-1644 / 5575) (Desulfotomaculum acetoxidans)* \| gram-negative \| \| *Enterobacteriaceae bacterium (strain FGI 57)* \| gram-negative \| \| *Enterococcus phoeniculicola ATCC BAA-412* \| gram-positive \| \| *Escherichia albertii (strain TW07627)* \| gram-negative \| \| *Escherichia coli (strain K12)* \| gram-negative \| \| *Escherichia coli 113290* \| gram-negative \| \| *Escherichia coli 1303* \| gram-negative \| \| *Escherichia coli M718* \| gram-negative \| \| *Escherichia coli O127:H6 (strain E2348/69 / EPEC)* \| gram-negative \| \| *Escherichia fergusonii ECD227* \| gram-negative \| \| *Geobacillus thermoleovorans B23* \| gram-positive \| \| *Gloeocapsa sp. PCC 7428* \| gram-negative \| \| *Haemophilus haemolyticus M19501* \| gram-negative \| \| *Halalkalibacterium halodurans (strain ATCC BAA-125 / DSM 18197 / FERM 7344 / JCM 9153 / C-125) (Bacillus halodurans)* \| gram-positive \| \| *Haloarcula amylolytica JCM 13557* \| gram-negative \| \| *Haloarcula californiae ATCC 33799* \| gram-negative \| \| *Haloarcula hispanica (strain ATCC 33960 / DSM 4426 / JCM 8911 / NBRC 102182 / NCIMB 2187 / VKM B-1755)* \| gram-negative \| \| *Haloarcula hispanica N601* \| gram-negative \| \| *Haloarcula japonica (strain ATCC 49778 / DSM 6131 / JCM 7785 / NBRC 101032 / NCIMB 13157 / TR-1)* \| gram-negative \| \| *Haloarcula marismortui (strain ATCC 43049 / DSM 3752 / JCM 8966 / VKM B-1809) (Halobacterium marismortui)* \| gram-negative \| \| *Haloarcula sinaiiensis ATCC 33800* \| gram-negative \| \| *Haloarcula sp. CBA1115* \| gram-negative \| \| *Halobiforma lacisalsi AJ5* \| gram-negative \| \| *Haloferax elongans ATCC BAA-1513* \| gram-negative \| \| *Haloferax larsenii JCM 13917* \| gram-negative \| \| *Halopiger xanaduensis (strain DSM 18323 / JCM 14033 / SH-6)* \| gram-negative \| \| *Halorhabdus utahensis (strain DSM 12940 / JCM 11049 / AX-2)* \| gram-negative \| \| *Halothece sp. (strain PCC 7418) (Synechococcus sp. (strain PCC 7418))* \| \| \| *Kyrpidia tusciae (strain DSM 2912 / NBRC 15312 / T2) (Bacillus tusciae)* \| gram-positive \| \| *Lacticaseibacillus rhamnosus (strain LMS2-1)* \| gram-positive \| \| *Lactococcus lactis subsp. cremoris (Streptococcus cremoris)* \| gram-positive \| \| *Leminorella grimontii ATCC 33999 = DSM 5078* \| gram-negative \| \| *Leptothrix ochracea L12* \| gram-negative \| \| *Liquorilactobacillus sucicola DSM 21376 = JCM 15457* \| gram-positive \| \| *Lyngbya sp. (strain PCC 8106) (Lyngbya aestuarii (strain CCY9616))* \| gram-negative \| \| *marine sediment metagenome* \| gram-negative \| \| *Mariniradius saccharolyticus AK6* \| gram-negative \| \| *Martelella endophytica* \| gram-negative \| \| *Mesorhizobium opportunistum (strain LMG 24607 / HAMBI 3007 / WSM2075)* \| gram-negative \| \| *Mesorhizobium sp. LNHC221B00* \| gram-negative \| \| *Methanoculleus marisnigri (strain ATCC 35101 / DSM 1498 / JR1)* \| gram-negative \| \| *Microcystis aeruginosa (strain NIES-843 / IAM M-2473)* \| gram-positive \| \| *Microcystis aeruginosa PCC 9717* \| gram-positive \| \| *Microcystis aeruginosa PCC 9806* \| gram-positive \| \| *Microcystis aeruginosa PCC 9809* \| gram-positive \| \| *Moorena producens 3L* \| gram-negative \| \| *Morganella morganii SC01* \| gram-negative \| \| *Morganella morganii subsp. morganii KT* \| gram-negative \| \| *Natrialba asiatica (strain ATCC 700177 / DSM 12278 / JCM 9576 / FERM P-10747 / NBRC 102637 / 172P1)* \| gram-negative \| \| *Natrinema pallidum DSM 3751* \| gram-negative \| \| *Neosynechococcus sphagnicola sy1* \| gram-negative \| \| *Nitrospira defluvii* \| gram-negative \| \| *Nodularia spumigena CCY9414* \| gram-negative \| \| *Nostoc punctiforme (strain ATCC 29133 / PCC 73102)* \| gram-negative \| \| *Oceanicola granulosus (strain ATCC BAA-861 / DSM 15982 / KCTC 12143 / HTCC2516)* \| gram-negative \| \| *Oligella urethralis DNF00040* \| gram-negative \| \| *Paenibacillus riograndensis SBR5* \| gram-positive \| \| *Pandoraea thiooxydans* \| gram-negative \| \| *Pantoea sp. (strain At-9b)* \| gram-negative \| \| *Pantoea sp. aB* \| gram-negative \| \| *Pantoea sp. GM01* \| gram-negative \| \| *Pantoea stewartii subsp. stewartii DC283* \| gram-negative \| \| *Pantoea vagans (strain C9-1) (Pantoea agglomerans (strain C9-1))* \| gram-negative \| \| *Paraburkholderia phytofirmans (strain DSM 17436 / LMG 22146 / PsJN) (Burkholderia phytofirmans)* \| gram-negative \| \| *Parasutterella excrementihominis CAG:233* \| gram-negative \| \| *Pectobacterium betavasculorum* \| gram-negative \| \| *Pectobacterium odoriferum* \| gram-negative \| \| *Pectobacterium parmentieri* \| gram-negative \| \| *Pectobacterium peruviense* \| gram-negative \| \| *Photorhabdus aegyptia* \| gram-negative \| \| *Photorhabdus laumondii subsp. laumondii (strain DSM 15139 / CIP 105565 / TT01) (Photorhabdus luminescens subsp. laumondii)* \| gram-negative \| \| *Photorhabdus luminescens (Xenorhabdus luminescens)* \| gram-negative \| \| *Photorhabdus temperata J3* \| gram-negative \| \| *Photorhabdus temperata subsp. temperata Meg1* \| gram-negative \| \| *Porphyromonas gingivicanis* \| gram-negative \| \| *Prevotella sp. CAG:755* \| gram-negative \| \| *Providencia rustigianii DSM 4541* \| gram-negative \| \| *Pseudodesulfovibrio aespoeensis (strain ATCC 700646 / DSM 10631 / Aspo-2) (Desulfovibrio aespoeensis)* \| gram-negative \| \| *Pusillimonas sp. (strain T7-7)* \| gram-negative \| \| *Rahnella aquatilis HX2* \| gram-negative \| \| *Rahnella sp. (strain Y9602)* \| gram-negative \| \| *Rhizobium meliloti (strain 1021) (Ensifer meliloti) (Sinorhizobium meliloti)* \| gram-negative \| \| *Salinisphaera hydrothermalis (strain C41B8)* \| gram-negative \| \| *Salmonella agona (strain SL483)* \| gram-negative \| \| *Salmonella arizonae (strain ATCC BAA-731 / CDC346-86 / RSK2980)* \| gram-negative \| \| *Salmonella bongori (strain ATCC 43975 / DSM 13772 / NCTC 12419)* \| gram-negative \| \| *Salmonella bongori N268-08* \| gram-negative \| \| *Salmonella choleraesuis (strain SC-B67)* \| gram-negative \| \| *Salmonella enterica subsp. arizonae* \| gram-negative \| \| *Salmonella enterica subsp. enterica serovar Alachua str. R6-377* \| gram-negative \| \| *Salmonella enterica subsp. enterica serovar Cubana str. 76814* \| gram-negative \| \| *Salmonella enterica subsp. enterica serovar Dublin str. UC16* \| gram-negative \| \| *Salmonella enterica subsp. enterica serovar Inverness str. R8-3668* \| gram-negative \| \| *Salmonella enterica subsp. enterica serovar Montevideo str. S5-403* \| gram-negative \| \| *Salmonella enterica subsp. enterica serovar Rubislaw str. A4-653* \| gram-negative \| \| *Salmonella enterica subsp. enterica serovar Senftenberg str. A4-543* \| gram-negative \| \| *Salmonella enterica subsp. enterica serovar Wandsworth str. A4-580* \| gram-negative \| \| *Salmonella enterica subsp. indica serovar 6,14,25:z10:1,(2),7 str. 1121* \| gram-negative \| \| *Salmonella gallinarum* \| gram-negative \| \| *Salmonella gallinarum (strain 287/91 / NCTC 13346)* \| gram-negative \| \| *Salmonella newport (strain SL254)* \| gram-negative \| \| *Salmonella paratyphi A (strain ATCC 9150 / SARB42)* \| gram-negative \| \| *Salmonella paratyphi C (strain RKS4594)* \| gram-negative \| \| *Salmonella typhi* \| gram-negative \| \| *Salmonella typhimurium* \| gram-negative \| \| *Salmonella typhimurium (strain 14028s / SGSC 2262)* \| gram-negative \| \| *Salmonella typhimurium (strain 4/74)* \| gram-negative \| \| *Salmonella typhimurium (strain LT2 / SGSC1412 / ATCC 700720)* \| gram-negative \| \| *Salmonella typhimurium (strain SL1344)* \| gram-negative \| \| *Serratia plymuthica S13* \| gram-negative \| \| *Serratia sp. Ag1* \| gram-negative \| \| *Sinorhizobium medicae (strain WSM419) (Ensifer medicae)* \| gram-negative \| \| *Sinorhizobium meliloti (strain BL225C)* \| gram-negative \| \| *Sinorhizobium meliloti (strain SM11)* \| gram-negative \| \| *Sinorhizobium meliloti CCNWSX0020* \| gram-negative \| \| *Skermanella stibiiresistens SB22* \| gram-negative \| \| *Sphingobium chlorophenolicum* \| gram-negative \| \| *Sphingobium chlorophenolicum L-1* \| gram-negative \| \| *Sphingobium quisquiliarum P25* \| gram-negative \| \| *Streptococcus thermophilus* \| gram-positive \| \| *Thermacetogenium phaeum (strain ATCC BAA-254 / DSM 26808 / PB)* \| gram-positive \| \| *Thermaerobacter subterraneus DSM 13965* \| gram-positive \| \| *Truepera radiovictrix (strain DSM 17093 / CIP 108686 / LMG 22925 / RQ-24)* \| gram-positive \| \| *Xenococcus sp. PCC 7305* \| gram-negative \| \| *Yokenella regensburgei ATCC 43003* \| gram-negative \| |
| --- | --- | --- | --- | --- | --- | --- | --- | --- | --- | --- | --- | --- | --- | --- | --- | --- | --- | --- | --- | --- | --- | --- | --- | --- | --- | --- | --- | --- | --- | --- | --- | --- | --- | --- | --- | --- | --- | --- | --- | --- | --- | --- | --- | --- | --- | --- | --- | --- | --- | --- | --- | --- | --- | --- | --- | --- | --- | --- | --- | --- | --- | --- | --- | --- | --- | --- | --- | --- | --- | --- | --- | --- | --- | --- | --- | --- | --- | --- | --- | --- | --- | --- | --- | --- | --- | --- | --- | --- | --- | --- | --- | --- | --- | --- | --- | --- | --- | --- | --- | --- | --- | --- | --- | --- | --- | --- | --- | --- | --- | --- | --- | --- | --- | --- | --- | --- | --- | --- | --- | --- | --- | --- | --- | --- | --- | --- | --- | --- | --- | --- | --- | --- | --- | --- | --- | --- | --- | --- | --- | --- | --- | --- | --- | --- | --- | --- | --- | --- | --- | --- | --- | --- | --- | --- | --- | --- | --- | --- | --- | --- | --- | --- | --- | --- | --- | --- | --- | --- | --- | --- | --- | --- | --- | --- | --- | --- | --- | --- | --- | --- | --- | --- | --- | --- | --- | --- | --- | --- | --- | --- | --- | --- | --- | --- | --- | --- | --- | --- | --- | --- | --- | --- | --- | --- | --- | --- | --- | --- | --- | --- | --- | --- | --- | --- | --- | --- | --- | --- | --- | --- | --- | --- | --- | --- | --- | --- | --- | --- | --- | --- | --- | --- | --- | --- | --- | --- | --- | --- | --- | --- | --- | --- | --- | --- | --- | --- | --- | --- | --- | --- | --- | --- | --- | --- | --- | --- | --- | --- | --- | --- | --- | --- | --- | --- | --- | --- | --- | --- | --- | --- | --- | --- | --- | --- | --- | --- | --- | --- | --- | --- | --- | --- | --- | --- | --- | --- | --- | --- | --- | --- | --- | --- | --- | --- | --- | --- | --- | --- | --- | --- | --- | --- | --- | --- | --- | --- | --- | --- | --- | --- | --- | --- | --- | --- | --- | --- | --- | --- | --- | --- | --- | --- | --- | --- | --- | --- | --- | --- | --- | --- | --- | --- | --- | --- | --- | --- | --- | --- | --- | --- | --- | --- | --- | --- | --- | --- | --- | --- | --- | --- | --- | --- | --- | --- | --- | --- | --- | --- | --- | --- | --- | --- | --- | --- | --- | --- | --- | --- | --- | --- |
